# Testing PET-[ ^11^ C]ABP688 as a tool to quantify glutamate release *in vivo*

**DOI:** 10.1162/imag_a_00126

**Published:** 2024-04-05

**Authors:** Hussein Bdair, Marie Sato-Fitoussi, Stéphane Planche, Luc Moquin, Min Su Kang, Arturo Aliaga, Atsuko Nagano-Saito, Kelly Smart, Sylvia M.L. Cox, Jamie Near, Argel Aguilar-Valles, Gassan Massarweh, Pedro Rosa-Neto, Chawki Benkelfat, Jean-Paul Soucy, Alexey Kostikov, Alain Gratton, Marco Leyton

**Affiliations:** Department of Psychiatry, McGill University, Montreal, Canada; Department of Neurology and Neurosurgery, Montreal Neurological Institute-Hospital, Montreal, Canada; Department of Psychiatry, Douglas Mental Health University Institute, McGill University, Montreal, Canada; Translational Neuroimaging Laboratory, McGill University Research Centre for Studies in Aging, Alzheimer’s Disease Research Unit, Douglas Research Institute, Montreal, Quebec, Canada; Department of Neurosciences, Health Sciences building, Carleton University, Ottawa, Canada; Department of Chemistry, McGill University, Montreal, Canada

**Keywords:** metabotropic glutamate type 5 (mGlu5) receptors, positron emission tomography, magnetic resonance spectroscopy, *in vivo* microdialysis, ethanol, transmitter release

## Abstract

The excitatory neurotransmitter glutamate plays a critical role inexperience-dependent neuroplasticity, including addiction-related processes. Todate, however, it is not possible to measure glutamate release in the livinghuman brain. Positron emission tomography (PET) with [^11^C]ABP688, aselective allosteric antagonist of metabotropic type 5 glutamate (mGlu5)receptors, could offer an effective strategy. To test this proposition, weconducted a series of studies in rats using microdialysis and[^11^C]ABP688 microPET imaging, and in humans using PET and magneticresonance spectroscopy (MRS). Significant calcium-dependent glutamate releasewas identified in the ventral striatum of awake rats (190.5 ± 34.7%,*p*< 0.05;*n*= 7) followingadministration of a low dose of ethanol (EtOH; 20%, 0.5 g/kg), a pharmacologicalchallenge readily translatable to human research. Simultaneous microdialysis andmicroPET studies in anesthetized rats yielded concurrent increases in glutamaterelease (126.9 ± 5.3%,*p *< 0.001;*n*= 11) and decreases in striatal[^11^C]ABP688 binding (6.8 ± 9.6%,*p <*0.05). These latter two effects, however, were not significantlycorrelated (*r*= 0.25,*p*= 0.46).In humans, a laboratory stressor yielded significant changes in self-reportedmood (*p*s < 0.041), sympathetic system activations(*p*s < 0.042), and the MRS index of striatalglutamate reuptake following excitatory neurotransmission, Glx/Cr levels(*p*= 0.048). These effects, however, were notaccompanied by significant changes in [^11^C]ABP688 BP_ND_(*p*s > 0.21,*n*= 9) orcorrelated with each other (*p*s > 0.074). Together, thesestudies document EtOH-induced glutamate release from neurons, EtOH-induceddecreases in [^11^C]ABP688 binding, and stress-induced changes inglutamate turnover, yet fail to provide evidence that the PET[^11^C]ABP688 method can be exploited to quantify moderate changes inglutamate release. The results underscore the need for highly controlled testingconditions during PET measures of mGlu5 receptors.

## 1 Introduction

Glutamate is the primary excitatory neurotransmitter in mammalian brain where itplays a central role in the effects of drugs and alcohol, drug withdrawal, andexperience-dependent neuroplasticity (Dahchour& De Witte, 2003;Fliegel et al.,2013;Saal et al., 2003;Stuber et al., 2008;Vigneault et al., 2015). Based on these observations,medications targeting glutamate neurotransmission are in development for variousneuropsychiatric disorders, including schizophrenia, depression, addictions, andfragile X syndrome (Dolen & Bear,2008;Goff & Coyle, 2001;Hashimoto et al., 2007;Kalivas, 2009;Levenga etal., 2011). Despite these advances, a lack of tools has hampered ourability to measure glutamate features that might be specific to humans and humanbrain-based diseases.

To date, several aspects of glutamate neurotransmission in the living human brain,including changes in glutamate levels, have only been assessed indirectly.Functional imaging techniques such as blood oxygen level dependent-functionalmagnetic resonance imaging (BOLD-fMRI) and[^18^F]fluorodeoxyglucose/positron emission tomography([^18^F]FDG/PET) can identify brain regional changes in activity, but theirsignals are not transmitter-specific (Knutson& Gibbs, 2007;Shafiei et al.,2019). Proton magnetic resonance spectroscopy (^1^H-MRS) canmeasure stress and pain-induced changes in glutamate turnover rate in apre-specified region of interest (ROI) (Bryant etal., 2013;Gutzeit et al., 2013),but these spectroscopic signals are difficult to interpret and are believed toprimarily indicate intracellular (neuronal and glial) levels, making the relation toneurotransmission uncertain (Ramadan et al.,2013).

A recently proposed alternative approach is to use positron emission tomography (PET)with [^11^C]ABP688 [3-((6-methylpyridin-2-yl)ethynyl)cyclohex-2-en-1-one-*O*-[^11^C]methyloxime], a tracerthat binds with high selectivity to the allosteric site of metabotropic type 5glutamate (mGlu5) receptors (Ametamey et al.,2006;Scala et al., 2021).PET/[^11^C]ABP688 studies have identified alterations in mGlu5 receptoravailability in populations with various psychiatric and neurodegenerativedisorders, including a major depressive disorder, substance use disorders, epilepsy,and frontotemporal dementia (Choi et al.,2014;Cox et al., 2020;Kim et al., 2019;Leuzy et al., 2016;Martinez et al., 2014;Milella et al.,2014;Smart et al., 2017).

Preliminary evidence also indicates that, in both laboratory animals and humans,[^11^C]ABP688 binding is affected by interventions that increase (DeLorenzo et al., 2015;Esterlis et al., 2018) and decrease (Zimmer et al., 2015) extracellular glutamateconcentrations. One possibility is that the changes in tracer binding are caused byglutamate release-inducing conformational changes that alter affinity at theallosteric binding site. Alternatively, since the reductions can remain for up to 24h, a glutamate surge-induced receptor internalization is also possible. Indeed, thismechanism is thought to account for long-lasting decreases in the binding ofD_2_receptor radioligands, such as [^11^C]raclopride and[^123^I]IBZM, following dopamine release (Laruelle, 2012;Laruelleet al., 1997). Finally, [^11^C]ABP688 binding values can vary byup to 70% when repeated PET scans are conducted on the same day. These changes mightreflect circadian rhythm related influences on both mGlu5 receptor expression (DeLorenzo et al., 2017;Elmenhorst et al., 2016) and glutamate release (Marquez de Prado et al., 2000) or,alternatively, diminished stress-induced glutamate release during the second scan(DeLorenzo et al., 2017;Lupinsky et al., 2010,^2017^). In either scenario, these findings together raise the possibilitythat changes in extracellular glutamate concentrations affect mGlu5 tracer bindingsystematically, thereby providing a non-invasive measure of glutamate release.

The gold standard for validating a PET measure of altered extracellularneurotransmitter levels is demonstrating that changes in PET tracer binding are*proportional*to changes in extracellular transmitterconcentration using*in vivo*microdialysis (Breier et al., 1997;Laruelle, 2012;Laruelle et al.,1997). To apply these techniques to PET-[^11^C]ABP688, aglutamate-release enhancer is required. Several animal studies have tentativelydemonstrated that moderately high doses of aqueous ethanol (EtOH) at a 20%concentration can promote neuronal dose-dependent glutamate release in the ventralstriatum in rodents (Fliegel et al., 2013).If confirmed at a dose more suitable for human ingestion, EtOH would be an easilyaccessible and administered glutamate trigger, fostering the translation ofpreclinical findings to clinical research.

Based on these observations, we first tested whether a low dose (0.5 g/kg) of 20%EtOH would lead to a glutamatergic response in the ventral striatum, as assessedwith*in vivo*microdialysis. Second, we measured the correlationbetween changes in EtOH-induced glutamate concentrations in the ventral striatum andalterations in striatal [^11^C]ABP688 non-displaceable binding potential(BP_ND_) values using simultaneous*in vivo*microdialysis and microPET. Finally, we examined, in healthy humans, the effect ofacute stress on [^11^C]ABP688 BP_ND_values, as measured with PETin mGlu5 receptor dense regions (Shigemoto et al.,1993). This latter work was combined with MRS measures of the effect ofacute stress on glutamate and glutamine levels in regions with reportedstress-induced alterations in glutamate–glutamine balance (Auer et al., 2000;Shethet al., 2019;Ullmann et al.,2020). The associations between [^11^C]ABP688 BP_ND_valuesand brain tissue glutamate and glutamine levels were then assessed. A PET ligandsensitive to proportionate changes in endogenous glutamate release in humans wouldbe a valuable new tool.

## 2 Methods

### 2.1 [ ^11^ C]ABP688 radiochemistry

>99% diastereomerically pure*E*-isomer,(*E*)-[^11^C]ABP688 was produced as previouslydescribed by our group (Bdair et al.,2019).

### 2.2 Animal study

All experimental designs were approved by McGill University Animal Care Committee(UACC; Animal Use Protocol # MNI-7914), in compliance with the guidelines of theCanadian Council on Animal Care (CCAC).

#### 2.2.1 Subjects

Male Lewis rats at age 60–65 postnatal days (PND) were purchased fromCharles River (Saint Constant, QC, Canada) and housed at the DouglasResearch Centre Animal Facility or the Center of Neurological Disease Models(CNDM) / McGill University, under 12-h/12-h light/dark cycle (lights open at7:00 am) in a stress- and noise-free environment. Water and a protein-richlaboratory chow diet were provided*ad libitum*in theircages. A maximum of two rats were housed in a single standard cage, suppliedwith a standard environmental enrichment. After arrival, the rats were leftto acclimate for at least seven days before use. Lewis strain was selectedbecause it exhibits stronger characteristic neurochemical and behavioralaspects of drug-seeking behavior (Cadoni,2016).

#### 2.2.2 Cannulation

Anesthesia was induced in rats inside an induction chamber using 4–5%isoflurane in medical air admixture at a flow rate of 2 L/min. Theanesthesia was maintained with 2–2.5 isoflurane at the same flow, andrats were then placed on a stereotaxic bed with a pre-warmed pad. The eyeswere treated with artificial tears to prevent ocular dryness, the scalp wasshaved, and its skin was injected with 0.1 mL of 5 mg/mL bupivacaineintradermally to desensitize the scalp. An incision was made to the scalpusing a sharp medical blade, and the skull was cleaned with hydrogenperoxide (H_2_O_2_). The target location of the cannula inthe left ventral striatum (anteroposterior; AP: +1.20 mm,mediolateral; ML: −1.40 mm) was determined following Paxinoscoordinates (Paxinos & Watson,2005). With a handheld drill, apertures for the cannula and threeanchor screws were made and the screws were the threaded in the cranium. A22-gauge quartz guide cannula was inserted 6.00 mm along the dorsoventral(DV) axis and fixed to the cranium using superglue followed by acrylicdental cement. The guide cannula was then capped with a stainless-steelobturator that extends 2.50 mm beyond the end of the cannula to preventinfection and cerebral spinal fluid (CSF) leakage. Post-operatively, therats were then injected subcutaneously with 20 g/kg of 0.9% NaCl (for oneday), and with 2.5 mg/kg carprofen (for 3 consecutive days) to compensatefor the lost blood and provide post-operative analgesia, respectively. Atopical antibiotic (Polysporin^®^) was applied to the woundand the animals were left for at least a week for recovery, housed one percage, before testing.

#### 2.2.3 In vivo microdialysis

##### 2.2.3.1 Standalone microdialysis

**Fig. 1.**
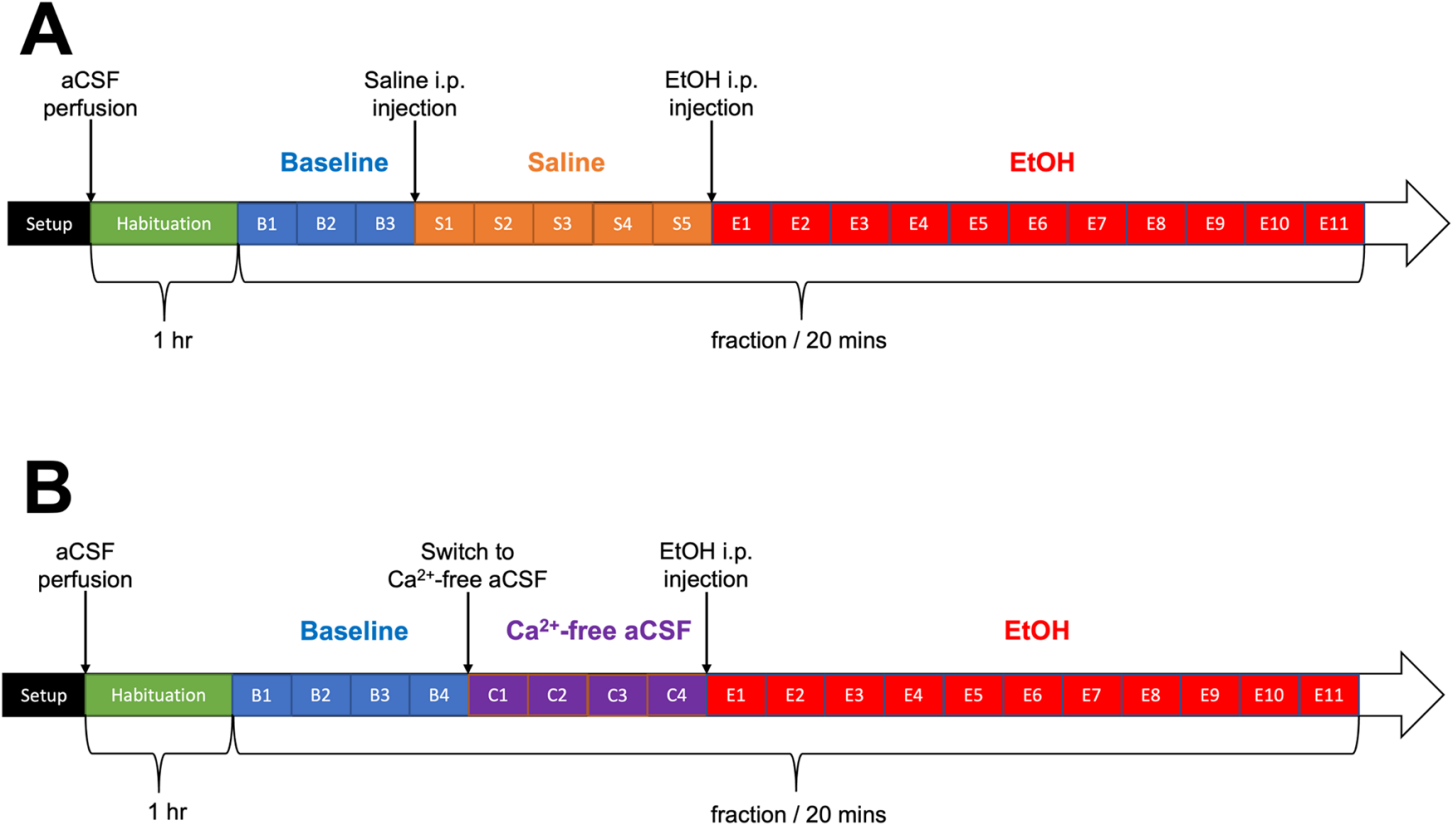
Diagrams of the microdialysis study using either (A) artificialcerebrospinal fluid (aCSF; n = 7) or (B) calcium-deprivedaCSF (*n*= 4) in awake animals.

*In vivo*microdialysis was initially conducted as astandalone technique in awake, freely moving rats (*n*= 7). Following a 20-min habituation period, the microdialysisprobe was inserted into the guide cannula and connected from the otherend to a Hamilton syringe filled with artificial cerebral spinal fluid(aCSF) and secured to a computer-controlled microinfusion pump (CMA).The aCSF (26 mM NaHCO_3_, 1.2 mM NaH_2_PO_4_,1.3 mM MgCl_2_, 2.3 mM CaCl_2_, 3.0 mM KCl, 126 mMNaCl, 0.2 mM L-ascorbic acid) was pumped to the probe at a flow rate of1 µL/min for a minimum of 1 h to stabilize neurotransmitterlevels. Samples were then collected at 20-min intervals, mixed with 1µL of 0.25 M perchloric acid, and stored in−80^°^C refrigerator for subsequent HPLCanalyses. Following the collection of three baseline dialysatefractions, the animals received an intraperitoneal (i.p.) injection of0.5 g/kg saline and five dialysate fractions were collected, followed byi.p. injection of 0.5 g/kg 20% EtOH with the collection of elevendialysate fractions (Fig. 1A).

Furthermore, to study the calcium dependency of the hypothesizedEtOH-induced glutamate release, the experiment was repeated with the useof a Ca^2+^-free aCSF instead (*n*= 4). In the latter, CaCl_2_was replaced with anequimolar concentration of MgCl_2_(final concentration: 3.6mM). The paradigm was similar to the EtOH challenge microdialysisdescribed above. However, after collecting four baseline samples, theperfusate was switched from aCSF to Ca^2+^-free aCSFusing a liquid switch (CMA), following which four fractions werecollected. After the animal received an i.p. injection of 0.5 g/kg 20%EtOH, eleven fractions were collected (Fig. 1B).

##### 2.2.3.2 Simultaneous microdialysis and microPET

*In vivo*microdialysis was conducted simultaneously withmicroPET scanning in anesthetized animals (*n*=11). Herein, animals were induced in a chamber with 4–5%isoflurane in medical air admixture and then maintained with1.5–2.5% isoflurane at 0.8 L/min flow rate through a nose cone.The rats were placed in prone position on the prewarmed bed of themicroPET scanner (CTI, Concorde Microsystems, LLC). Each rat underwenttwo microdialysis/microPET scans on separate days of < 7 daysapart, where the animal received i.p. injection of 0.5 g/kg of eithersaline or 20% EtOH.

As in the standalone microdialysis study, aCSF was pumped at a flow rateof 1 µL/min and dialysate fractions were collected every 20 min.Subsequent to the collection of four baseline dialysate fractions, ratsreceived an i.p. injection of the pharmacological intervention (i.e.,saline or 20% EtOH). The collection of first post-saline or -EtOHdialysate sample started at the moment of injection. The radiotracer wasinjected intravenously 5 min post-saline or -EtOH injection. The*simultaneous*microdialysis and microPET studydesign is depicted inFigure 2.

**Fig. 2.**
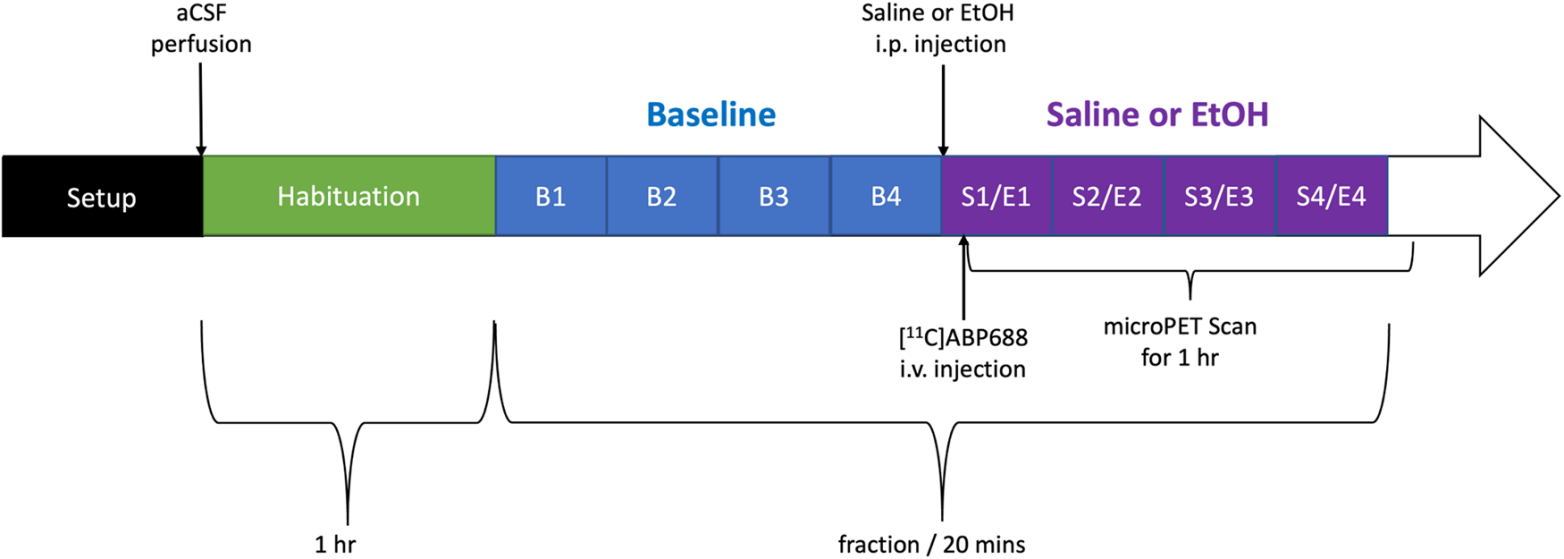
Diagram of the simultaneous microdialysis plus microPETstudy.

#### 2.2.4 Analysis of dialysate samples

Measurements of dialysate glutamate concentrations were determined with anHPLC precolumn derivatization with ultimate 3000 RS fluorescence detection(ex: 322 nm; emission: 455 nm) and further described byLupinsky et al. (2010). The HPLC system consistedof a Dionex pump (ultimate 3000) and a Dionex RS autosampler (ultimate 3000)bundled with a Waters Xterra MS C18 3.0 × 50 mm 5 μmanalytical column. The mobile phase is 3.5% MeCN, 20% methanol(CH_3_OH), and 100 mmol/L sodium phosphate dibasic(Na_2_HPO_4_) adjusted to pH 6.7 with 85% phosphoricacid (H_3_PO_4_). The flow rate was set at0.5 mL/min. Working standards (100 ng/mL) and derivatizationreagents were prepared fresh daily and loaded with samples into arefrigerated (10 °C) Dionex RS autosampler (ultimate 3000). Beforeinjection onto the analytical column, each fraction was sequentially mixedwith 20 μL of o-phthaldehyde (0.0143 mol/L) diluted with 0.1 mol/Lsodium tetraborate and 20 μL of 3-mercaptopropionic acid (0.071mol/L) diluted with H_2_O and allowed to react for 10 min.After each injection, the injection loop was flushed with 20%CH_3_OH to prevent contamination of subsequent samples. Under theseconditions, the retention time for glutamate was approximately 1 minwith a total run time of 30 min/sample.

#### 2.2.5 Animal PET scanning

All rats (*n*= 11) underwent two microPET scans,baseline and challenge, conducted between 11:00 and 13:00 (to mitigatepotential effects of circadian glutamate variations). The microPET procedurewas conducted in the anesthetized rats using microPET R4 scanner (CTI,Concorde Microsystems, LLC; spatial resolution of approximately 1.85 mm(Knoess et al., 2003)). Aftercollecting a minimum of 4 baseline dialysate samples, 0.5 g/kg of eithersaline (baseline) or 20% EtOH (challenge) was injected intraperitoneally,and the rats were placed in the scanner’s center field of view (FOV).Five minutes after EtOH or saline injection, a 0.5–1.0 mL bolusinjection of (*E*)-[^11^C]ABP688 at an average doseof 21.83 MBq (range: 19.94–24.98 MBq) was administered intravenouslyin the lateral tail vein through a pre-inserted catheter, followed by a60-min dynamic emission acquisition. A total of 27 frames were acquired (9× 30 s, 6 × 1 min, 5 × 2 min, 7 × 5 min),followed by a 9-min transmission scan using a rotating^57^Cosource.

#### 2.2.6 Animal MRI scanning

Magnetic resonance imaging (MRI) structural images for co-registrationpurposes were obtained using a 7T Bruker Pharmascan pre-clinical MRI system,with a Bruker volume resonator radiofrequency (RF) coil designed for ratbrain imaging. Two-dimensional T2-weighted MRI images were obtained withmulti-slice TurboRARE acquisition, in-plane resolution of 0.2 × 0.2mm^2^, and slice thickness of 0.5 mm. The sequence included aTR of 6000 ms, TEeff of 30 ms, RARE factor of 4, 42 slices covering thewhole rat brain, 20 signal averages, and total acquisition time of 42 min.The in-plane FOV was 2.50 cm × 3.50 cm.

#### 2.2.7 Image processing and analyses

Images were reconstructed using*Maximum a Posteriori*(MAP)algorithm with scatter correction, then processed, and analyzed using MINCtoolkit software (http://bic-mni.github.io). Co-registration of PET and MRI imageswas performed with MINC toolkit using both eyes, olfactory bulbs, temporalpoles, and base of the skull as registration landmarks. Atlas-basedauto-segmentation of various ROIs was performed with ITK-SNAP (v. 3.6.0;http://www.itksnap.org/pmwiki/pmwiki.php) (Yushkevich et al., 2006), based on brain regiondelineations reported in Waxholm Space atlas of the Sprague Dawley rat brain(Papp et al., 2014), where theMRI images were linearly co-registered to the atlas. Mean BP_ND_ofROIs was calculated relative to the non-specific binding in cerebellum,using the simple reference tissue model (SRTM) (Gunn et al., 1997), as previously reported (Elmenhorst et al., 2010). Percentchange in [^11^C]ABP688 BP_ND_was calculated asfollowing: ((BP_ND BASELINE_– BP_ND CHALLENGE_)/BP_ND BASELINE_) × 100.

### 2.3 Human study

#### 2.3.1 Participants

Healthy, right-handed volunteers (5 males and 4 females) aged 25.1 ±6.0 (Mean ± SD) years old were recruited from the general populationusing online advertisements on the McGill University website and throughclassified advertisements. Exclusion criteria included: (1) current or pastDSM-5 disorders, including current or past substance use except foroccasional cannabis use (< once per month), social tobacco use(< once per week), and occasional drinking (≤ seven drinks perweek); (2) family history of DSM-5 disorder; (3) current or past chronicmedication use, excluding birth control; (4) significant physical illness inthe past 12 months; (5) any history of head injury/loss of consciousness;(6) any counterindications to MRI or PET including claustrophobia, and thepresence of a medical condition that makes pain stimuli dangerous (e.g.,cardiac disease, hypertension, pulmonary disease, seizure disorder,osteopenia, and anxiety syndromes). The study protocol was approved by theResearch Ethics Board of the Montreal Neurological Institute and the Facultyof Medicine and was carried out in accordance with the Declaration ofHelsinki. Next, physical health was evaluated by a routine examination, astandard blood work, and electrocardiogram. A urine toxicology test forillicit drugs of abuse (Triage, Biosite Diagnostics, San Diego, CA,responsive to amphetamines, methamphetamines, barbiturates, benzodiazepines,cocaine, 2-ethylene-1,5-dimethyl-3,3-diphenylpyrrolidine (EDDP), opiates,Δ^9^-tetrahydrocannabinol (Δ^9^-THC),and tricyclic antidepressants) and a urine pregnancy test for women wasperformed on the screening day and prior to each PET session.

#### 2.3.2 Stress administration

**Fig. 3.**
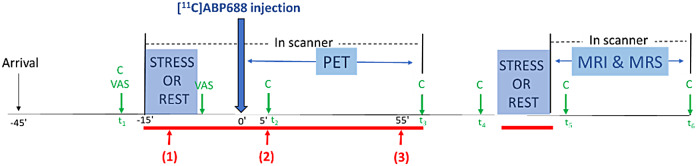
Timing of human test sessions. X-axis denotes time relative toinjection at time 0. Participants underwent a stress or rest task 15min before injection of the PET tracer and starting the MR scan.Mood and physiological data were collected throughout. The red barsrepresent periods when electrodermal activity was tracked. VAS= visual analog scale; C = cortisol measurement.

All participants underwent two scanning sessions consisting of a 1-h PET scanfollowed by a 45 min MRI scan with MRS. The two scanning days were conductedin a counter-balanced, within-subjects cross-over design, at least 3 daysapart, with each PET and MR scan conducted at the same time of the day tomitigate potential effects of circadian glutamate variations (Fig. 3).

**Fig. 4.**
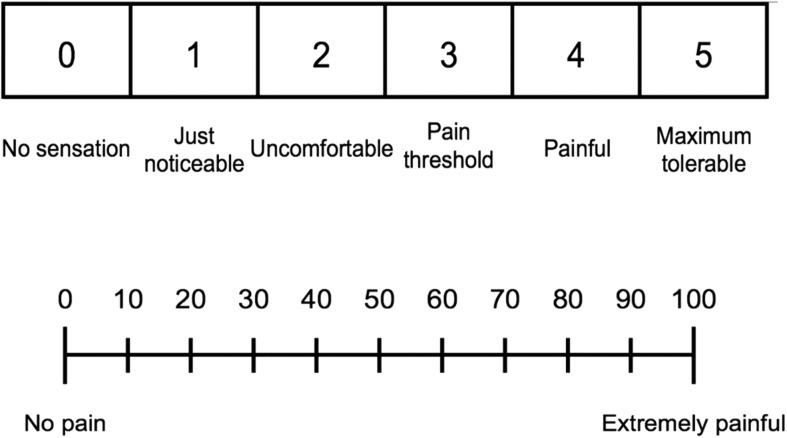
Pain scale (top) and pain visual analog scale (bottom).

The acute stress stimulus consisted of unpredictable electrical stimulationof the wrist administered immediately below the individual’s painthreshold, defined as the lowest intensity at which a sensation of mild painis felt. Participants observed 20 s countdowns followed by a blank screenduring which electric stimulation occurred 67% of the time,pseudo-randomized. After a 10 s rest, the paradigm was repeated. In 6 min,participants experienced 12 × 30 s blocks, for a total of eightelectrical stimulations out of 12 trials. Participants were instructed priorto the stress task that they would receive intermittent electricalstimulation at the level of their threshold. Participants were not given adistinguishing cue to identify whether a stimulation would be followed by agiven countdown nor were they informed of the contingency rate. Participantsprovided verbal ratings of discomfort on the pain scale and visual analogscale (VAS;Fig. 4) every time thestimulus was presented, permitting adjustments to the intensity.

The pain threshold was determined outside of the PET scanner environment asfollows. Electric stimulation was initiated with a duration of 200 ms andvoltage of 20 V and was increased in increments of 2 V until the lowestvoltage at which subjects experienced moderate discomfort was reached. Thiswas defined as a score of 3 on the 5-point pain scale and at least 20 on theVAS. The threshold was re-established on the stress session, immediatelyprior to the stress task. The pain threshold determined earlier was used asa starting point to minimize the number of shocks administered before thetask. The intensity of shock corresponding to the threshold was then used asthe target intensity for the stimulation during the stress task.

#### 2.3.3 Objective indices of stress response

To assess the effectiveness of the stimuli, variation of the electricalconductance in response to skin secretion was tracked continuouslythroughout stress/rest tasks and PET scanning sessions. First, phasicdeflections in the skin conductance responses (SCRs) were analyzed (Pitman & Orr, 1986). The SCRamplitudes during the stress task were calculated by subtracting the meanskin conductance level 2 s before expectation of a shock, from the peakvalue obtained immediately after administration of the shocks. To avoidhabituation (Davis, 1934), phasicincreases occurring over the first five trials of shocks were taken intoaccount. The same calculation was applied over the first three periods ofblank screens in which shock was expected but not triggered. During the sametime interval at rest, amplitudes of non-specific SCRs (occurring in theabsence of stimuli) were calculated. Lastly, tonic skin conductance wascompared: three time intervals were averaged, including (1) stress task orrest, (2) the first 5 to 10 min of PET, and (3) the last 5 min of PET (Fig. 3). The time interval (2)corresponds to the peak of the hypothalamus-pituitary-adrenal (HPA) axisresponse in response to stress, which is expected to begin 20 min after theinitiation of the stimulus (t2).

The HPA axis response to stress was assessed by measuring cortisolconcentrations from saliva samples collected using oral swabs (Salimetrics,LLC). In total, six saliva samples were collected over 120 min: at baseline(t_1_and t_4_), at two time points after initiationof the task (t_2_and t_5_: 20 min after initiation of thetask), and at the end of each scan (t_3_and t_6_). Areaunder the curve with respect to ground (AUC_G_), and with respectto increase from cortisol value at t_1_(AUC_I_) werecalculated as described (Pruessner et al.,2003). AUC_G_is the total area under the curve, whereasAUC_I_is calculated with reference to the first value(cortisol value at t_1_). Samples were stored at −20°C until biochemical analysis took place.

#### 2.3.4 Behavioral assessment

Subjective ratings of mood, anxiety, and alertness were measured using thestate-trait anxiety inventory (STAI)-State (Spielberger, 1983), and visual analog scale of alertness. Eachscale was collected two times each session, before and immediately after thefirst stress task (Fig. 3).

#### 2.3.5 Human PET scanning

All PET scans were performed using a High-Resolution Research Tomograph(HRRT; Siemens/CTI, Knoxville, TN, USA) at the Montreal NeurologicalInstitute. Scans consisted of a 60-min dynamic acquisition collected inlist-mode format, followed by a 6-min^137^Cs rotating point sourcetransmission scan for attenuation correction. The acquisition was binnedinto frames, the durations of which consisted of the following sequence: 3× 10 s, 5 × 30 s, 4 × 60 s, 4 × 120 s, 5× 300 s, 2 × 60 s. The scan initiated concurrently with thebeginning of the venous injection of an average dose of 385.54 MBq (range:333–407 MBq) of (*E*)-[^11^C]ABP688 throughan intravenous catheter installed at the participant’s right arm vein(antecubital region).

#### 2.3.6 Human MRI and MRS scanning

For PET/MR co-registration and spectroscopy voxel placement, all subjectsalso underwent a high-resolution T1-weighted MRI scan after each PETsession. Scans were acquired in a 3T Siemens TRIO Magneton scanner (SiemensMedical Solutions, Erlangen, Germany) using an ADNI-3D MPRAGE protocol.Images were acquired in 3D repetition time (TR) = 2300 ms, echo time(TE) = 3.42 ms, flip angle = 9°, field of view =256 mm, and FOV = 256 × 256; 1 mm resolution isotropicresolution.

During the same session, MR spectroscopy scanning was conducted in twovolumes of interests from which measures of combined glutamate (Glu) andglutamine (Gln), referred to as Glx, and Glu alone were obtained.Spectroscopic voxels were prescribed from anatomic images: a 20 × 15× 10 mm^3^voxel was placed bilaterally over the anteriorcingulate cortex (ACC), immediately anterior to the rostrum of the corpuscallosum, and perpendicular to the infra-callosal line. The striatum voxelwas 25 × 12 × 12 mm^3^in size, encompassing theright dorsal caudate-putamen. The water-suppressed proton spectra wereacquired using a 90˚–180˚–180˚ (PRESS)sequence (TR = 3000 ms, TE = 40 ms), giving a total of 196acquisitions. A water-unsuppressed reference scan to enable correction foreddy current-induced phase shifts was obtained immediately after thewater-suppressed scan using the same TR, TE, voxel position, and shimsettings with 16 acquisitions.

#### 2.3.7 Image processing and analyses

CIVET pipeline (https://www.bic.mni.mcgill.ca/ServicesSoftware/CIVET) was used topreprocess the native MRI image. The resampled images were then classifiedinto white matter (WM), grey matter (GM), and CSF; segmented in the mainbrain structures; and automatically labeled, using the ANIMAL probabilisticatlas-based algorithm (Collins et al.,1999). Then, the PET images were co-registered with thesubject’s own MRI transformed into the Montreal NeurologicalInstitute (MNI) template brain using transformed parameters obtained fromthe registration of MRI to MNI152 space. Mean BP_ND_of ROI wasestimated using SRTM (Gunn et al.,1997), with the cerebellar grey matter as reference region.Subcortical limbic regions amygdala and hippocampus were yielded by thesegmentation generated by the ANIMAL image registration algorithm. Astandard mask was used to functionally segment the striatum into ventral(VST), associative (AST), and sensorimotor (SMST) subregions, as proposed byMawlawi et al. (2001). Remainingcortical ROIs were manually drawn on a template MRI in stereotaxic spaceusing the software DISPLAY (http://www.bic.mni.mcgill.ca/software/Display/Display.html) andwere based on the approach defined byAbi-Dargham et al. (2000). Percent change in[^11^C]ABP688 BP_ND_was calculated as following:((BP_ND STRESS_– BP_ND REST_)/ BP_NDREST_) × 100.

### 2.4 Statistical analyses

Analyses were performed using SPSS software. (Version 29). Differences wereconsidered statistically significant at*p*< 0.05.Shapiro–Wilk tests established normal distribution of all data.

For standalone microdialysis study, the effects of EtOH administration weretested using a one-way repeated-measures analysis of variance (rmANOVA) withtime as the within-group factor containing 17 levels (pooled baseline and eachsubsequent fraction; five saline samples and eleven EtOH samples). Averagevalues were extracted for each condition (baseline: B1-3; saline: S1-5; EtOH:E1-6) and compared using a one-way rmANOVA with condition as the within-groupfactor containing three levels. Both ANOVAs contained tests for violations ofsphericity; Huynh-Feldt corrections were applied when necessary.Bonferroni-corrected paired t-tests were then used to compare the threeexperimental conditions.

In the simultaneous microdialysis and microPET study, a two-way within-subjectrmANOVA test was performed to study the effect of treatment (saline vs. EtOH)over time (five levels; pooled baseline and four subsequent posttreatmentfraction) on extracellular glutamate levels in the ventral striatum, as assessedwith microdialysis. Regarding PET images, a one sample (i.e., paired) t-test wasconducted to determine the percent difference in [^11^C]ABP688BP_ND_in the ventral striatum. Pearson’s*r*test was then used to examine the correlation between the percent differencebetween extracellular glutamate concentrations (saline vs. EtOH) and[^11^C]ABP688 displacement. Percent change of individual dialysatefraction (averaged across all animals) was calculated relative to pooledbaseline fractions. Peak percent of baseline is defined as the dialysatefraction with the highest extracellular glutamate concentration compared topooled baseline fractions.

For the human study, the effect of stress on subjective anxiety and physiologicalmeasurements (cortisol and IL1- β) were identified using two-way rmANOVAsor mixed-model analyses when data were missing, with sessions (rest vs. stress)and timepoints as within-subject factors. Simple-main effects analyses followedwhen indicated. Planned pair-wise t-tests were carried out to identifydifferences in the magnitude of SCR and non-stimuli SCR relative to thenon-specific SCRs. Summary BP_ND_values were computed as theunweighted mean of all examined regions in order to assess the effects of tracerand scan characteristics (mass of tracer injected per kilogram body weight andtime of injection). Relationships between BP_ND_and scanscharacteristics were assessed using Pearson’s*r.*To testthe main hypothesis of differences in BP_ND_between conditions,separate Condition x Region x Hemisphere repeated-measures ANOVAs were performedfor (i) striatal regions (VST, AST, SMST), (ii) prefrontal regions (medial(mPFC), dorsolateral (dlPFC) prefrontal cortices, orbitofrontal cortex (OFC),and ACC), and (iii) limbic regions (amygdala and hippocampus). These werefollowed by planned, uncorrected two-tailed dependent measures t-tests to assesseach contrast in the selected ROIs between conditions. For each ROI, percentchange from scan 1 to scan 2 ((BP_ND STRESS_– BP_NDREST_)/ BP_ND REST_× 100%) was calculated for eachparticipant. Parametric maps of BP_ND_were compared in voxel-wisepaired t-tests from scan 1 to scan 2 in each participant using RMINC with asignificance threshold of*p *< 0.05, corrected for falsediscovery rate. To determine the significance of detected metabolitesconcentration differences due to shock administration, a Condition (rest,stress) by Region (ACC, striatum) two-way rmANOVA was applied to the MRS data.Finally, potential associations of mGlu5 receptor availability with behavioraland physiological variables were examined using Pearson’s*r*. Given the large number of correlations performed, theunadjusted alpha level was divided by the number of studied ROIs, which resultedin a significance threshold of*p*= 0.05/9 =0.0056. In a secondary voxel-wise analysis, further exploratory correlationsusing mGlu5 binding across the whole brain were assessed with parameters whichrevealed to be significantly correlated with ROI-wise BP_ND_.

## 3 Results

### 3.1 Radiochemistry

The diastereomeric excess (d.e.) and radiochemical purity (RCP) of*(E)-*[^11^C]ABP688 were both >99%. Meanmolar activity (A_m_) at time of injection was 83.24 GBq/µmol(range: 28.46–268.28 GBq/µmol,*n*= 22) inthe animal study and 91.71 GBq/µmol (range: 23.1–163.6GBq/µmol;*n*= 18) in the human study.

### 3.2 *In vivo* microdialysis

**Fig. 5.**
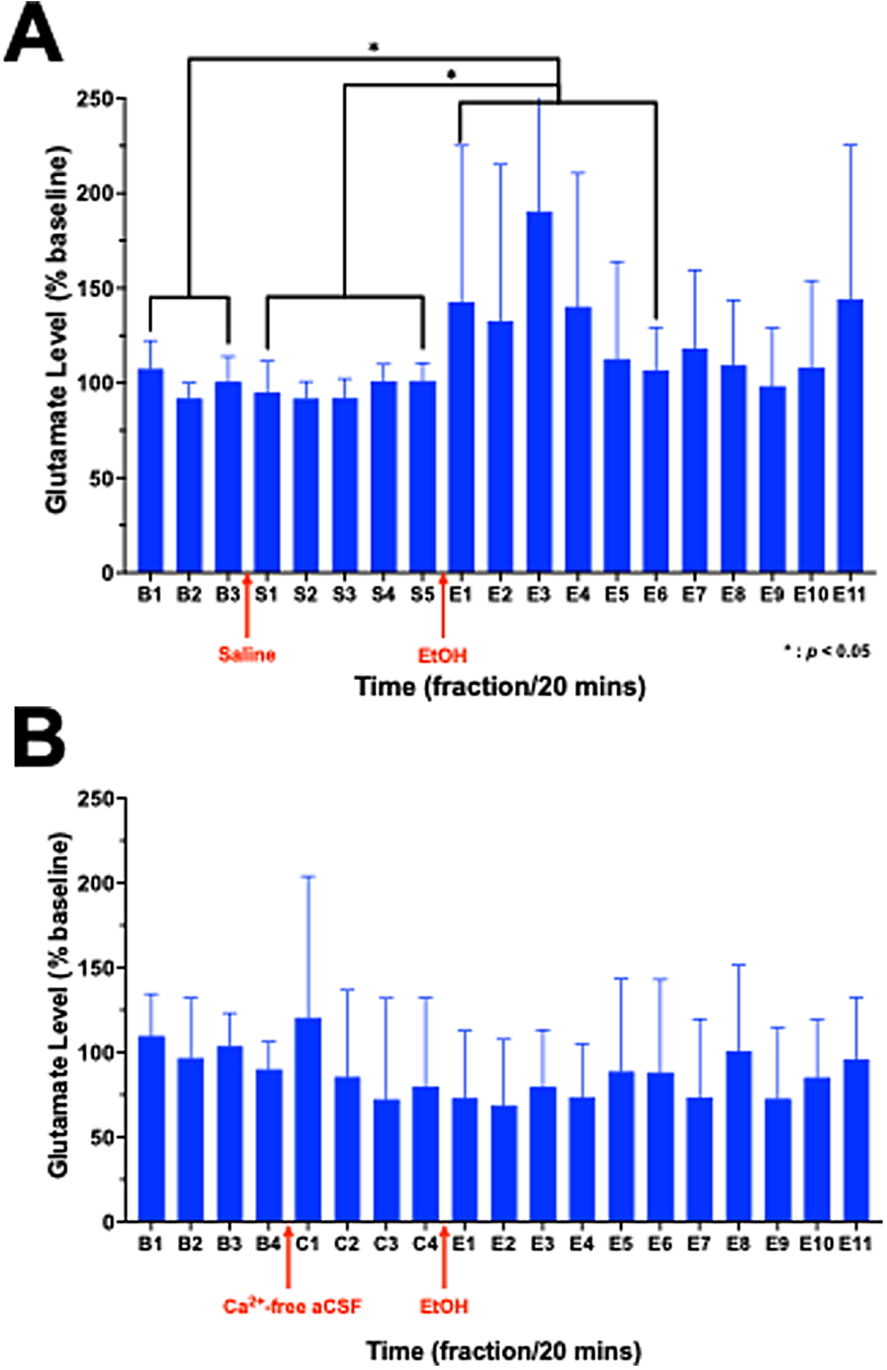
Percent change from baseline of extracellular glutamate concentrations inthe ventral striatum as measured by microdialysis in*awake*animals, following either (A) i.p. injectionof 0.5 g/kg saline and 0.5 g/kg 20% EtOH (*n*= 7)or (B) infusion of Ca^2+^-free aCSF and i.p. injectionof 0.5 g/kg 20% EtOH (*n*= 4). Values are Mean± SD. *p< 0.05.

As depicted inFigure 5A, there were maineffects of time (F(4.891,29.346) = 2.901,*p*=0.035) and condition (F(1.240,7.439) = 10.621,*p*= 0.010) in awake rats. This reflected significant increases inextracellular glutamate concentrations following EtOH administration (E1– E5) as compared to samples collected following the saline injection (S1– S5: t(6) = 3.322,*p*= 0.048) and thepre-injection samples (B1 – B3: t(6) = 3.372,*p*= 0.045). As expected, no differences were seen between the baseline andsaline conditions (*p*> 0.50). Compared to the averagesaline response, the largest effect occurred during the third dialysate samplecollected between 40–60 min after EtOH injection (E3) (t(6) =2.458, uncorrected*p*= 0.049). During this E3 fraction,extracellular glutamate concentrations reached 190.5 ± 34.7% of averagebaseline and 212.05 ± 47.52% of average saline. This effect of EtOH wasabolished when using the Ca^2+^-free aCSF (Fig. 5B).

**Fig. 6.**
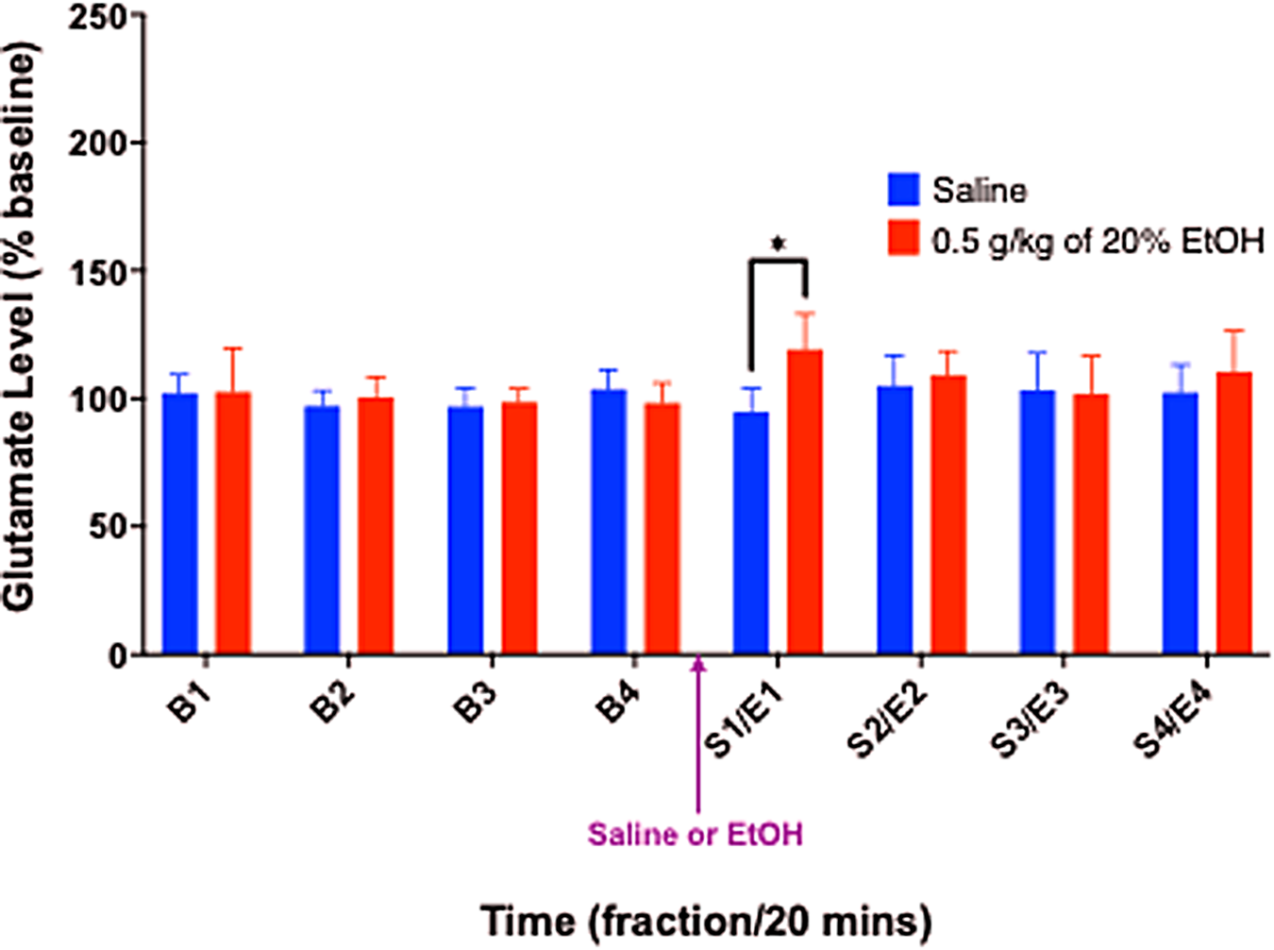
Percent change from baseline (B1-4) of extracellular glutamateconcentrations in the ventral striatum as measured by microdialysis,following i.p. injection of 0.5 g/kg of either saline or 20% EtOH in*anesthetized*animals (*n*=11). Values are Mean ± SD. *p<0.05

During the simultaneous microdialysis and microPET study in anesthetized rats, atwo-way rmANOVA yielded a significant treatment (saline vs. EtOH) by timeinteraction (F(2.505,25.046) = 3.353,*p*= 0.042;*n*= 11). Post-hoc pairwise comparisons revealed asignificant difference between the first two post-treatment dialysate fractionsfrom the pooled baseline (*p*s < 0.05, uncorrected), andthere was a significant difference between the effects of EtOH and saline in thefirst fraction (S1 vs. E1: (t(10) = –5.066,*p*< 0.001) reflecting concentrations that were 126.9 ± 5.3% ofbaseline (Fig. 6).

### 3.3 Animal PET scanning

The two PET scans did not significantly differ (*p*s >0.05) in the injected*(E)-*[^11^C]ABP688 dose(baseline: mean 22.24 MBq, range 20.72–24.98 MBq; challenge: mean 21.43MBq, range 19.94–24.98 MBq), A_m_(baseline: mean 55.79GBq/µmol, range 28.46–131.15 GBq/µmol; challenge: mean107.95 GBq/µmol, range 37.72–269.28 GBq/µmol), injectedvolume (baseline: mean 0.62 mL, range 0.5–1.0 mL; challenge: mean 0.58mL, range 0.5–0.75 mL), injected mass (baseline: mean 1.3 pmol/g, range0.5–2.3 pmol/g; challenge: mean 0.9 pmol/g, range 0.3–2.0 pmol/g),start time (baseline: mean 12:22, range 11:09–13:15; challenge: mean12:24, range 11:13–13:29), or weight of the animals (baseline: mean 306.8g, range 276–384 g; challenge: mean 311.3 g, range 266–349 g). Theaverage age of the animals was 83.5 ± 7.7 (Mean ± SD) at the timeof the baseline scan, and 87.8 ± 7.1 days (Mean ± SD) at the timeof the challenge scan.

**Fig. 7.**
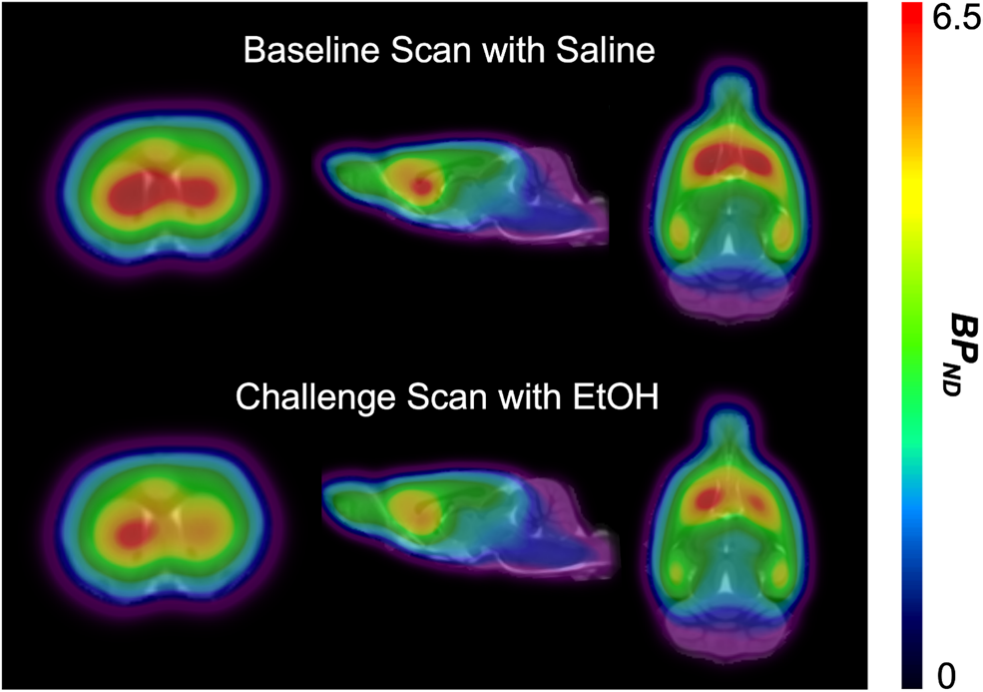
Average PET images in rodents (*n*= 11, coronal:left, sagittal: middle, axial: right) showing [^11^C]ABP688BP_ND_following i.p. injection of 0.5 g/kg of eithersaline (top) or 20% EtOH (bottom).

There was a significant percent reduction in striatal [^11^C]ABP688BP_ND_in response to EtOH administration compared to saline,corresponding to a percent change of 6.8 ± 9.6% (baselineBP_ND_: mean 4.78, range 4.18–5.83; challenge BP_ND_:mean 4.45, range 3.23–5.85; t(10) = 2.424,*p*= 0.036) (Fig. 7). The ratio ofpercent glutamate increase during E1 (compared to S1) to percent[^11^C]ABP688 BP_ND_reduction was 4:1. Percent changes inextracellular glutamate concentrations in the ventral striatum were notsignificantly correlated with percent changes in striatal [^11^C]ABP688BP_ND_(*r*= 0.25,*p*= 0.46).

### 3.4 Human study

#### 3.4.1 Physiological stress responses

**Fig. 8.**
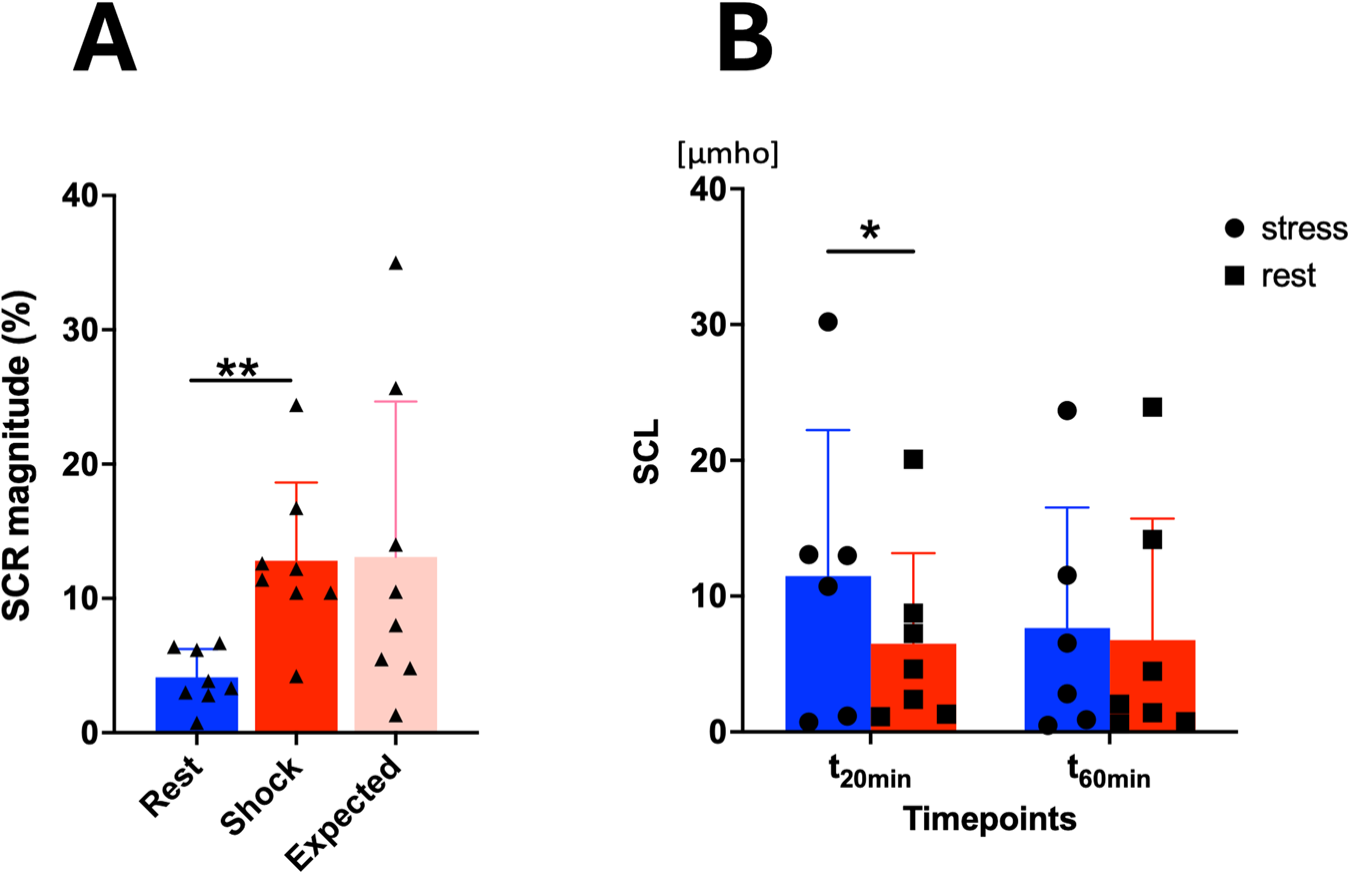
(A) The Skin Conductance Response was significantly higher duringshock exposure than rest. The peak that occurs when shocks wereexpected but not given also showed trend-level effects reflectinghigher peaks than at rest. (B) Twenty minutes after the initiationof shocks, SCL remained higher than at rest, but this effect did notpersist after an hour. Values are Mean ± SD. *p< 0.05; **p< 0.01

Skin conductance responses were significantly higher following exposure tothe stressor (t(7) = 4.65,*p*= 0.0023).Numerically greater increases also occurred when shocks were expected, butnot given, but this response was more variable and at the trend level only(t(7) = 2,*p*= 0.09) (Fig. 8). Twenty minutes following initiation of thetask, skin conductance levels remained elevated (t(7) = 3,*p*= 0.04) before normalizing after an hour (t(7)= 0.047,*p*= 0.96).

AUC_I_between stress and rest were significantly different (t(7)= 2,*p*= 0.041), reflecting increased versusdecreased cortisol levels during the stress (AUC_I STRESS_=6.97) versus rest session (AUC_I REST_= −6.83). Thestress-induced percent increases in cortisol and skin conductance levelswere significantly correlated (*r*= 0.838,*p*= 0.009).

#### 3.4.2 Self-report responses

**Fig. 9.**
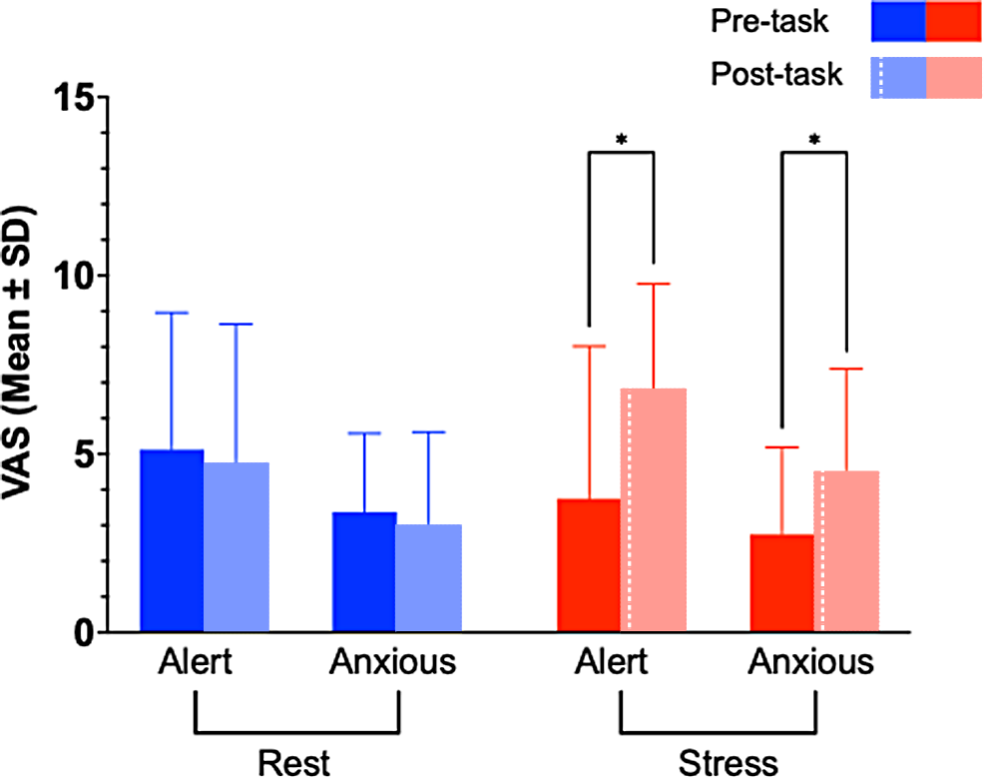
Anxiety and alertness ratings were significantly higher afteradministration of the stress task relative to before administrationand relative to baseline. Solid bars represent pre-task (–20min pre-injection), whereas white-hashed bars represent post-task(+20 min post-injection). Red indicates rest session; blueindicates stress session. Values are Mean ± SD. *p< 0.05.

Significant Condition × Timepoint interactions were obtained for the“alert” (F(1,7) = 9.471,*p*=0.018) and “anxious” VAS measures (F(1,7) = 6.25,*p*= 0.041), but not “afraid”(F(1,7) = 3.3,*p*= 0.11). Further inspectionof the data confirmed that in the stress condition, alertness and anxietyratings were higher post-stress (t2) relative to pre-stress (t1) (alert:t(7) = 3.784,*p*= 0.0137; anxious: t(7)= 3.005,*p*= 0.0396, respectively;Fig. 9).

#### 3.4.3 PET/MRS

The two PET test sessions did not differ in injected tracer dose (rest: mean378.88 MBq, range 355.2–392.2 MBq; stress: mean 388.87 MBq, range333–407 MBq; t(8) = −1.16,*p*=0.28), A_m_(rest: mean 89.32 GBq/µmol, range23.1–128 GBq/µmol; stress mean 94.1 GBq/µmol, range24–163.6 GBq/µmol; t(8) = 1.204,*p*= 0.26), or start time (rest: mean 12:30, range 11:07–15:03;stress: mean 12:37, range 11:03–14:08; t(8) = −0.28,*p*= 0.79). Global BP_ND_values werenot related to the mass of [^11^C]ABP688 injected(*r*= 0.22,*p*= 0.37) ortime of injection (*r*= 0.27,*p*= 0.79).

Three-way Condition x Subregion x Hemisphere repeated-measures ANOVAs did notidentify a significant main or interaction effect (Fs < 0.97,*p*s > 0.41). Controlling for test session order(stress session in the first scan vs. second scan) did not affect theresults. Likewise, replacing the “Condition” factor by the“Day” factor (first scan vs. second scan) did not change theresults. Percent change in BP_ND_was calculated and averagedacross all ROIs within a subject, which ranged from −17.5% to 18.6%.A global tendency of increase was found across regions between conditions,but this did not reach statistical significance in post-hoc pairwisecomparisons (*ps*> 0.3, uncorrected). Voxel-wiseparametric analyses were consistent with these findings, with no clusters ofsignificant voxels emerging.

**Fig. 10.**
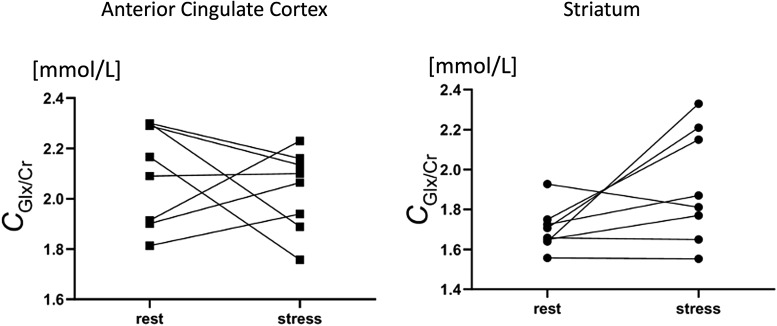
Changes from rest scan to stress scan in combined glutamate andglutamine concentrations (mmol/L) for each participant in theanterior cingulate cortex (squares) and striatum (circles).

Analysis of the combined Glutamate + Glutamine (Glx) levels did notyield a significant main effect of Session (F(1,7) = 1.09,*p*= 0.33) but a trend level Region x Sessioninteraction was seen (F(1,7) = 0.16,*p*=0.08). Post-hoc exploratory tests yielded evidence of significantstress-induced increases in Glx concentrations in the striatum (13%increase,*p*= 0.048) but not in the ACC (2%increase,*p*= 0.5;Fig. 10).

#### 3.4.4 Correlations

In humans, there were no significant associations between stress-inducedchanges in BP_ND_values and stress-induced changes in Glx/Gluratios or between BP_ND_values and Glx/Glu ratios at rest.However, BP_ND_values on the stress session (BP_NDSTRESS_) in limbic, sensorimotor, and associative striatum, OFC,and left amygdala were all negatively correlated with stress-induced changesin Glx/Glu levels in the ACC (*r*s > −0.71,*p*s < 0.044, uncorrected). BP_ND STRESS_values were also associated with Glx/Glu levels in the ACC at stress in theACC and both associative and sensorimotor striatum (*r*s> −0.43,*p*s < −0.71,*p*s < 0.048, uncorrected), and to a lesser extentin the hippocampus (*r*= −0.7,*p*= 0.054, uncorrected). Stress-induced changesin the cortisol AUC_G_were also correlated with BP_NDSTRESS_values in the striatum, OFC, amygdale, and hippocampus(*rs*< −0.72,*p*s <0.045, uncorrected). Correlations that survived at*p*= 0.0056 are shown inFigure11.

**Fig. 11.**
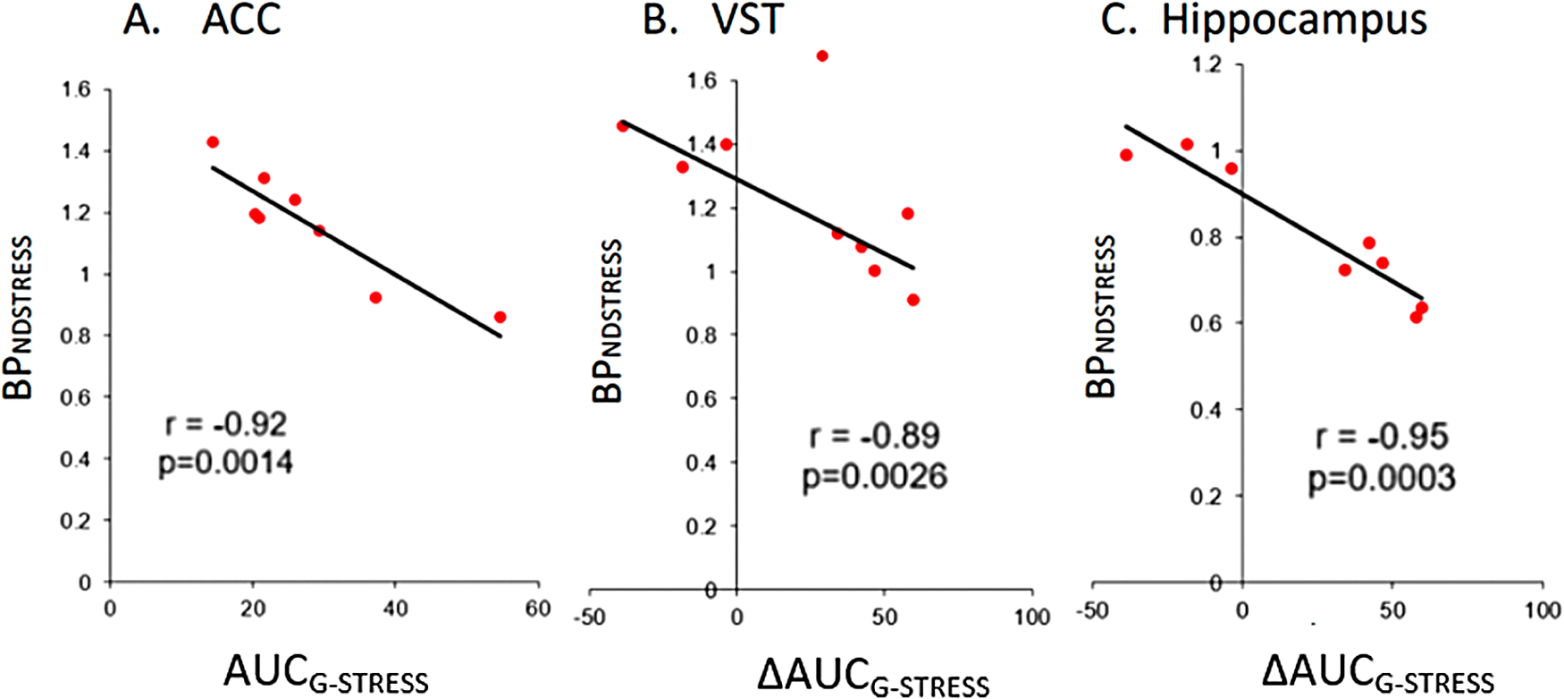
Association between mGlu5 receptor availability on the stress sessionand cortisol AUC_G-STRESS_response (A) and changes incortisol AUC_G_(B-C). (A) Anterior Cingulate Cortex (ACC).(B) Right ventral (limbic) striatum (VST). Regions that demonstratedsignificance at*p*≤ 0.0056 are shown.

## 4 Discussion

The present series of experiments yielded four novel findings. First, as predicted,administration of 0.5 g/kg of 20% EtOH led to a doubling of extracellular glutamateconcentrations within the ventral striatum. This contrasts with smaller glutamateeffects produced by higher EtOH doses, as predicted byFliegel et al. (2013). By extrapolating the trend lineconstructed from the ventral striatal glutamatergic responses to higher EtOH doses(1, 2, and 3 g/kg) (Fliegel et al., 2013),they hypothesized that a lower dose (i.e., 0.5 g/kg) would lead to a glutamatergicresponse of about 200%. Second, this glutamatergic effect of EtOH relies onCa^2+^-dependent exocytotic release, implicating a neuronalsource of the transmitter. Third, the low-dose EtOH challenge induced significantdecreases in striatal [^11^C]ABP688 BP_ND_values in rodents.Fourth, changes in BP_ND_values did not systematically covary with thechanges in dialysate glutamate concentrations in rats or MRS measured indices ofglutamate turnover in humans.

The mechanism by which low-dose EtOH induces a glutamatergic response is not fullyunderstood. One possibility is that EtOH acts as a negative modulator of theionotropic glutamatergic receptors,*N*-methyl-D-aspartate (NMDA).Drawing such a conclusion is plausible since ketamine, a non-competitive NMDAreceptor antagonist, was found to elicit a similar dose-dependent glutamatergiceffect to that proposed with EtOH (Moghaddam et al.,1997). Furthermore, there is evidence that NMDA receptor antagonism islinked to the activation of the glutamatergicα-amino-3-hydroxy-5-methyl-4-isoxazolepropionic acid (AMPA) receptors (Maeng et al., 2008)*via*predominately decreased activity of inhibitory γ-aminobutyric acid (GABA)interneurons (Homayoun & Moghaddam,2007).

Contrary to what was observed in awake animals, the glutamatergic responses inanesthetized rats peaked at approximately 127% of saline during the E1 fractioninstead of continuing to grow through to the E3 fraction. This curtailed responselikely reflects an effect of the anesthetic, isoflurane. Isoflurane is believed toabate excitatory glutamatergic transmission*via*decreasing synapticglutamate release (Larsen et al., 1998) andincreasing uptake by isolated nerve terminals (Larsen et al., 1997) and astrocytes (Miyazaki et al., 1997). The glutamate-suppressing effect of isofluranecould also explain why ketamine administration causes a significant reduction in[^11^C]ABP688 binding in humans (DeLorenzo et al., 2015;Esterlis et al.,2018), but not in anesthetized rodents (Kosten et al., 2018). Nevertheless, despite the relatively limitedincrease in EtOH-induced glutamate release in anesthetized rodents, we observed asignificant EtOH-induced reduction in striatal [^11^C]ABP688BP_ND_. This adds to the evidence that striatal [^11^C]ABP688binding responds to changes in glutamate concentrations with a microdialysis to PETratio of 4:1. This change compares quite favorably to the 44:1 and 64:1 ratios seenfor [^11^C]raclopride and dopamine release responses induced by 0.2 and 0.4mg/kg of*d*-amphetamine, respectively (Breier et al., 1997). Despite this, the percent change instriatal [^11^C]ABP688 BP_ND_did not correlate with changes inextracellular glutamate concentrations (*r*= 0.25,*p*= 0.46). This might be attributed to the smallincrease in EtOH-induced glutamate release (~27%), which in turn could have alimited effect on [^11^C]ABP688 displacement from mGlu5 receptors. Indeed,the percent change in EtOH-induced decreases in [^11^C]ABP688BP_ND_is in the range of test-retest variability for the striatumpreviously reported in rodents (Elmenhorst et al.,2012) (6.8% vs. 4.8%). In comparison,*d*-amphetamine (0.2and 0.4 mg/kg) induces larger effects on both [^11^C]raclopride binding(10.5% and 21.3%) and dopamine release (459% and 1365%), respectively (Breier et al., 1997).

Since [^11^C]ABP688 and glutamate do not share the same binding sites, ourmicrodialysis/microPET findings suggest that glutamate binding to the activeorthosteric site causes indirect displacement of the radiotracer from the allostericsite. It is hypothesized that glutamate binding to the orthosteric site causeseither alteration in the affinity of the allosteric site to [^11^C]ABP688or internalization of mGlu5 receptors, precluding the radiotracer from binding tothe allosteric site.

A limitation of our study is that the microdialysis measurements represent changes inventral striatum glutamate concentrations, whereas the PET signal represents thefull striatum. This noted,Fliegel et al.(2013)hypothesized that a low dose of EtOH (i.e., 0.5 g/kg) wouldincrease glutamate release throughout the entire striatum (i.e., ventral striatumand caudate-putamen). Future studies could also address whether the EtOH-inducedglutamate response from neurons reflects impulse-dependent activity; this could betested by measuring the effect of tetrodotoxin (TTX), an Na^+^channel blocker that inhibits the firing of action potentials (Lupinsky et al., 2010). A second limitation to our animalstudies is that only male rats were used. Future studies will be needed to examine[^11^C]ABP688 sensitivity to glutamate release in females.

In our study in humans, a series of electric shocks was sufficient to increasestriatal Glx:Glu ratios, putatively reflecting elevated glutamine metabolism (Yuksel & Ongur, 2010) followingstress-induced glutamate release, enhanced glial glutamate reuptake, and subsequentconversion to glutamine. However, these effects were not large. Potentially relatedto this, exposure to the laboratory stressor did not lead to significant changes in[^11^C]ABP688 BP_ND_values. This might reflect markedindividual differences in the responses rather than statistical noise. Indeed,exploratory analyses identified negative correlations between stress-inducedincreases in salivary cortisol and BP_ND_values in the amygdala, ACC, OFC,and limbic striatum on the stress session. Since, however, correlations were notseen with stress-induced changes in BP_ND_values, the above associationsshould be interpreted cautiously. Both men and women were tested, but the modestsample size precluded sufficient statistical power to test for a possible effect ofsex.

Although our study reduced time-related scan variability as much as possible, bothacross sessions and between individual participants, one participant underwent acontrol scan much later than the stress scan due to a tracer production failure.This same person exhibited higher binding at rest compared to other participants,which could be attributed to circadian rhythm effects, causing diurnal variations in[^11^C]ABP688 binding as previously reported in animal and humanstudies (DeLorenzo, Kumar, et al., 2011;Elmenhorst et al., 2016).

We found associations between [^11^C]ABP688 BP_ND_values on thestress session with ACC Glx:Glu values at stress, as well as with stress-inducedchanges in ACC Glx:Glu values. However, these correlations did not survivecorrection for multiple comparisons, somewhat limiting the generalizability of thefindings. Supporting this caution, mGlu5 receptor availability and MRS measures ofglutamate have been measured in cocaine-dependent and healthy subjects (Martinez et al., 2014) and correlations betweenthe two modalities were not found. In comparison, a second study identified acorrelation between MRS measured glutamate turnover in the ACC and[^18^F]FPEB binding, another PET tracer that binds to the same site as[^11^C]ABP688, in patients with major depression (Abdallah et al., 2017). However, these results likelyreflected long-term effects of elevated glutamate levels on receptor availability,an interpretation supported by post-mortem tissue evidence that patients with ahistory of depression have reduced mGlu5 protein expression (Deschwanden et al., 2011).

Lastly, BP_ND_values calculated using the cerebellum as a reference regioncorrelate highly with values derived with arterial input (Michele et al., 2024) but low levels of mGlu5 receptorspecific binding have been identified in the human cerebellum (Michele et al., 2024), raising the possibility that thiscould produce systematic biases (DeLorenzo, Milak,et al., 2011;Kagedal et al.,2013), potentially decreasing the ability to identify associations. Thishypothesized effect would not influence our rodent data since their cerebellum hasnegligible mGlu5 receptors (Ametamey et al.,2006;Kessler, 2004;Wyss et al., 2007).

## 5 Conclusion

The present study has demonstrated that a low dose (0.5g/kg) of 20% EtOH causes asignificant calcium-dependent increase in ventral striatum glutamate release inawake animals. Although the EtOH-induced glutamatergic response was less pronouncedin anesthetized rats, this smaller increase in glutamate release was accompanied bya significant reduction in striatal [^11^C]ABP688 BP_ND_with aratio of 4:1. Nevertheless, a significant correlation was not observed betweenchanges in ventral striatum glutamate release and striatal [^11^C]ABP688binding. Lastly, no correlation was observed between stress-induced changes inGlx/Glu and [^11^C]ABP688 BP_ND_in humans. Together, thesefindings suggest that [^11^C]ABP688 binding could be affected by moderatefluctuations in extracellular glutamate release but does not provide a proportionalmeasure. Future PET studies of mGlu5 receptor availability should use highlycontrolled testing conditions to avoid these effects.

## Data Availability

Data are available upon request from the corresponding author.

## References

[b1] Abdallah , C. G. , Hannestad , J. , Mason , G. F. , Holmes , S. E. , DellaGioia , N. , Sanacora , G. , Jiang , L. , Matuskey , D. , Satodiya , R. , Gasparini , F. , Lin , X. , Javitch , J. , Planeta , B. , Nabulsi , N. , Carson , R. E. , & Esterlis , I. ( 2017 ). Metabotropic glutamate receptor 5 and glutamate involvement in major depressive disorder: A multimodal imaging study . Biological Psychiatry. Cognitive Neuroscience and Neuroimaging , 2 ( 5 ), 449 – 456 . 10.1016/j.bpsc.2017.03.019 28993818 PMC5630181

[b2] Abi-Dargham , A. , Martinez , D. , Mawlawi , O. , Simpson , N. , Hwang , D. R. , Slifstein , M. , Anjilvel , S. , Pidcock , J. , Guo , N. N. , Lombardo , I. , Mann , J. J. , Van Heertum , R. , Foged , C. , Halldin , C. , & Laruelle , M. ( 2000 ). Measurement of striatal and extrastriatal dopamine D1 receptor binding potential with [11C]NNC 112 in humans: Validation and reproducibility . Journal of Cerebral Blood Flow and Metabolism , 20 ( 2 ), 225 – 243 . 10.1097/00004647-200002000-00003 10698059

[b3] Ametamey , S. M. , Kessler , L. J. , Honer , M. , Wyss , M. T. , Buck , A. , Hintermann , S. , Auberson , Y. P. , Gasparini , F. , & Schubiger , P. A. ( 2006 ). Radiosynthesis and preclinical evaluation of 11C-ABP688 as a probe for imaging the metabotropic glutamate receptor subtype 5 . Journal of Nuclear Medicine , 47 ( 4 ), 698 – 705 . https://pubmed.ncbi.nlm.nih.gov/16595505/ 16595505

[b4] Auer , D. P. , Putz , B. , Kraft , E. , Lipinski , B. , Schill , J. , & Holsboer , F. ( 2000 ). Reduced glutamate in the anterior cingulate cortex in depression: An in vivo proton magnetic resonance spectroscopy study . Biological Psychiatry , 47 ( 4 ), 305 – 313 . 10.1016/s0006-3223(99)00159-6 10686265

[b5] Bdair , H. , Tsai , I. H. , Smart , K. , Benkelfat , C. , Leyton , M. , & Kostikov , A. ( 2019 ). Radiosynthesis of the diastereomerically pure (E)-[11C]ABP688 . Journal of Labelled Compounds & Radiopharmaceuticals , 62 ( 12 ), 860 – 864 . 10.1002/jlcr.3802 31418468

[b6] Breier , A. , Su , T.-P. , Saunders , R. , Carson , R. E. , Kolachana , B. S. , de Bartolomeis , A. , Weinberger , D. R. , Weisenfeld , N. , Malhotra , A. K. , Eckelman , W. C. , & Pickar , D. ( 1997 ). Schizophrenia is associated with elevated amphetamine-induced synaptic dopamine concentrations: Evidence from a novel positron emission tomography method . Proceedings of the National Academy of Sciences of the United States of America , 94 ( 6 ), 2569 – 2574 . 10.1073/pnas.94.6.2569 9122236 PMC20129

[b7] Bryant , R. A. , Felmingham , K. L. , Das , P. , & Malhi , G. S. ( 2013 ). The effect of perceiving control on glutamatergic function and tolerating stress . Molecular Psychiatry , 19 , 533 . 10.1038/mp.2013.60 23670487

[b8] Cadoni , C. ( 2016 ). Fischer 344 and Lewis rat strains as a model of genetic vulnerability to drug addiction . Frontiers in Neuroscience , 10 ( 13 ), 13 . 10.3389/fnins.2016.00013 26903787 PMC4746315

[b9] Choi , H. , Kim , Y. K. , Oh , S. W. , Im , H. J. , Hwang , D. W. , Kang , H. , Lee , B. , Lee , Y.-S. , Jeong , J. M. , Kim , E. E. , Chung , J.-K. , & Lee , D. S. ( 2014 ). In vivo imaging of mGluR5 changes during epileptogenesis using [11C]ABP688 PET in pilocarpine-induced epilepsy rat model . PLoS One , 9 ( 3 ), e92765 . 10.1371/journal.pone.0092765 24663806 PMC3963947

[b10] Collins , D. L. , Zijdenbos , A. P. , Baaré , W. F. , & Evans , A. C. ( 1999 ). ANIMAL+ INSECT: Improved cortical structure segmentation . Paper presented at the Information Processing in Medical Imaging: 16th International Conference, IPMI’99 Visegrád , Hungary , June 28–July 2, 1999 Proceedings 16. 10.1007/3-540-48714-x_16

[b11] Cox , S. M. L. , Tippler , M. , Jaworska , N. , Smart , K. , Castellanos-Ryan , N. , Durand , F. , Allard , D. , Benkelfat , C. , Parent , S. , Dagher , A. , Vitaro , F. , Boivin , M. , Pihl , R. O. , Côté , S. , Tremblay , R. E. , Séguin , J. R. , & Leyton , M. ( 2020 ). mGlu5 receptor availability in youth at risk for addictions: Effects of vulnerability traits and cannabis use . Neuropsychopharmacology , 45 ( 11 ), 1817 – 1825 . 10.1038/s41386-020-0708-x 32413893 PMC7608187

[b12] Dahchour , A. , & De Witte , P. ( 2003 ). Excitatory and inhibitory amino acid changes during repeated episodes of ethanol withdrawal: An in vivo microdialysis study . European Journal of Pharmacology , 459 ( 2–3 ), 171 – 178 . 10.1016/S0014-2999(02)02851-0 12524143

[b13] Davis , R. C. ( 1934 ). Modification of the galvanic reflex by daily repetition of a stimulus . Journal of Experimental Psychology , 17 ( 4 ), 504 – 535 . 10.1037/h0074305

[b14] DeLorenzo , C. , DellaGioia , N. , Bloch , M. , Sanacora , G. , Nabulsi , N. , Abdallah , C. , Yang , J. , Wen , R. , Mann , J. J. , Krystal , J. H. , Parsey , R. V. , Carson , R. E. , & Esterlis , I. ( 2015 ). In vivo ketamine-induced changes in [11C]ABP688 binding to metabotropic glutamate receptor subtype 5 . Biological Psychiatry , 77 ( 3 ), 266 – 275 . 10.1016/j.biopsych.2014.06.024 25156701 PMC4277907

[b15] DeLorenzo , C. , Gallezot , J. D. , Gardus , J. , Yang , J. , Planeta , B. , Nabulsi , N. , Ogden , R. T. , Labaree , D. C. , Huang , Y. H. , Mann , J. J. , Gasparini , F. , Lin , X. , Javitch , J. A. , Parsey , R. V. , Carson , R. E. , & Esterlis , I. ( 2017 ). In vivo variation in same-day estimates of metabotropic glutamate receptor subtype 5 binding using [11C]ABP688 and [18F]FPEB . Journal of Cerebral Blood Flow and Metabolism , 37 ( 8 ), 2716 – 2727 . 10.1177/0271678x16673646 27742888 PMC5536783

[b16] DeLorenzo , C. , Kumar , J. S. , Mann , J. J. , & Parsey , R. V. ( 2011 ). In vivo variation in metabotropic glutamate receptor subtype 5 binding using positron emission tomography and [11C]ABP688 . Journal of Cerebral Blood Flow and Metabolism , 31 ( 11 ), 2169 – 2180 . 10.1038/jcbfm.2011.105 21792244 PMC3210337

[b17] DeLorenzo , C. , Milak , M. S. , Brennan , K. G. , Kumar , J. S. , Mann , J. J. , & Parsey , R. V. ( 2011 ). In vivo positron emission tomography imaging with [11C]ABP688: Binding variability and specificity for the metabotropic glutamate receptor subtype 5 in baboons . European Journal of Nuclear Medicine and Molecular Imaging , 38 ( 6 ), 1083 – 1094 . 10.1007/s00259-010-1723-7 21279350 PMC3095762

[b18] Deschwanden , A. , Karolewicz , B. , Feyissa , A. M. , Treyer , V. , Ametamey , S. M. , Johayem , A. , Burger , C. , Auberson , Y. P. , Sovago , J. , Stockmeier , C. A. , Buck , A. , & Hasler , G. ( 2011 ). Reduced metabotropic glutamate receptor 5 density in major depression determined by [11C]ABP688 PET and postmortem study . American Journal of Psychiatry , 168 ( 7 ), 727 – 734 . 10.1176/appi.ajp.2011.09111607 21498461 PMC3129412

[b19] Dolen , G. , & Bear , M. F. ( 2008 ). Role for metabotropic glutamate receptor 5 (mGluR5) in the pathogenesis of fragile X syndrome . Journal of Physiology , 586 ( 6 ), 1503 – 1508 . 10.1113/jphysiol.2008.150722 18202092 PMC2375688

[b20] Elmenhorst , D. , Aliaga , A. , Bauer , A. , & Rosa-Neto , P. ( 2012 ). Test-retest stability of cerebral mGluR5 quantification using [11C]ABP688 and positron emission tomography in rats . Synapse , 66 ( 6 ), 552 – 560 . 10.1002/syn.21542 22290765

[b21] Elmenhorst , D. , Mertens , K. , Kroll , T. , Oskamp , A. , Ermert , J. , Elmenhorst , E. M. , Wedekind , F. , Beer , S. , Coenen , H. H. , & Bauer , A. ( 2016 ). Circadian variation of metabotropic glutamate receptor 5 availability in the rat brain . Journal of Sleep Research , 25 ( 6 ), 754 – 761 . 10.1111/jsr.12432 27357735

[b22] Elmenhorst , D. , Minuzzi , L. , Aliaga , A. , Rowley , J. , Massarweh , G. , Diksic , M. , Bauer , A. , & Rosa-Neto , P. ( 2010 ). In vivo and in vitro validation of reference tissue models for the mGluR5 ligand [11C]ABP688 . Journal of Cerebral Blood Flow and Metabolism , 30 ( 8 ), 1538 – 1549 . 10.1038/jcbfm.2010.65 20531460 PMC2949244

[b23] Esterlis , I. , DellaGioia , N. , Pietrzak , R. H. , Matuskey , D. , Nabulsi , N. , Abdallah , C. G. , Yang , J. , Pittenger , C. , Sanacora , G. , Krystal , J. H. , Parsey , R. V. , Carson , R. E. , & DeLorenzo , C. ( 2018 ). Ketamine-induced reduction in mGluR5 availability is associated with an antidepressant response: An [11C]ABP688 and PET imaging study in depression . Molecular Psychiatry , 23 ( 4 ), 824 – 832 . 10.1038/mp.2017.58 28397841 PMC5636649

[b24] Fliegel , S. , Brand , I. , Spanagel , R. , & Noori , H. R. ( 2013 ). Ethanol-induced alterations of amino acids measured by in vivo microdialysis in rats: A meta-analysis . In Silico Pharmacol , 1 ( 1 ), 7 . 10.1186/2193-9616-1-7 25505652 PMC4230485

[b25] Goff , D. C. , & Coyle , J. T. ( 2001 ). The emerging role of glutamate in the pathophysiology and treatment of schizophrenia . American Journal of Psychiatry , 158 ( 9 ), 1367 – 1377 . 10.1176/appi.ajp.158.9.1367 11532718

[b26] Gunn , R. N. , Lammertsma , A. A. , Hume , S. P. , & Cunningham , V. J. ( 1997 ). Parametric imaging of ligand-receptor binding in PET using a simplified reference region model . Neuroimage , 6 ( 4 ), 279 – 287 . 10.1006/nimg.1997.0303 9417971

[b27] Gutzeit , A. , Meier , D. , Froehlich , J. M. , Hergan , K. , Kos , S. , C , V. W. , Weymarn , C. V. , Lutz , K. , Ettlin , D. , Binkert , C. A. , Mutschler , J. , Sartoretti-Schefer , S. , & Brugger , M. ( 2013 ). Differential NMR spectroscopy reactions of anterior/posterior and right/left insular subdivisions due to acute dental pain . European Radiology , 23 ( 2 ), 450 – 460 . 10.1007/s00330-012-2621-0 22968781

[b28] Hashimoto , K. , Sawa , A. , & Iyo , M. ( 2007 ). Increased levels of glutamate in brains from patients with mood disorders . Biological Psychiatry , 62 ( 11 ), 1310 – 1316 . 10.1016/j.biopsych.2007.03.017 17574216

[b29] Homayoun , H. , & Moghaddam , B. ( 2007 ). NMDA receptor hypofunction produces opposite effects on prefrontal cortex interneurons and pyramidal neurons . Journal of Neuroscience , 27 ( 43 ), 11496 – 11500 . 10.1523/jneurosci.2213-07.2007 17959792 PMC2954603

[b30] Kagedal , M. , Cselenyi , Z. , Nyberg , S. , Raboisson , P. , Stahle , L. , Stenkrona , P. , Varnäs , K. , Halldin , C. , Hooker , A. C. , & Karlsson , M. O. ( 2013 ). A positron emission tomography study in healthy volunteers to estimate mGluR5 receptor occupancy of AZD2066—Estimating occupancy in the absence of a reference region . Neuroimage , 82 , 160 – 169 . 10.1016/j.neuroimage.2013.05.006 23668965

[b31] Kalivas , P. W. ( 2009 ). The glutamate homeostasis hypothesis of addiction . Nature Reviews: Neuroscience , 10 ( 8 ), 561 – 572 . 10.1038/nrn2515 19571793

[b32] Kessler , L. J. ( 2004 ). Development of novel ligands for PET imaging of the metabotropic glutamate receptor subtype 5 (mGluR5) . ETH Zurich . 10.3929/ethz-a-004842638

[b33] Kim , J. H. , Joo , Y. H. , Son , Y. D. , Kim , J. H. , Kim , Y. K. , Kim , H. K. , Kim , H.-K. , Lee , S.-Y. , & Ido , T. ( 2019 ). In vivo metabotropic glutamate receptor 5 availability-associated functional connectivity alterations in drug-naive young adults with major depression . European Neuropsychopharmacology , 29 ( 2 ), 278 – 290 . 10.1016/j.euroneuro.2018.12.001 30553696

[b34] Knoess , C. , Siegel , S. , Smith , A. , Newport , D. , Richerzhagen , N. , Winkeler , A. , Jacobs , A. , Goble , R. N. , Graf , R. , Wienhard , K. , & Heiss , W. D. ( 2003 ). Performance evaluation of the microPET R4 PET scanner for rodents . European Journal of Nuclear Medicine and Molecular Imaging , 30 ( 5 ), 737 – 747 . 10.1007/s00259-002-1052-6 12536244

[b35] Knutson , B. , & Gibbs , S. E. ( 2007 ). Linking nucleus accumbens dopamine and blood oxygenation . Psychopharmacology , 191 ( 3 ), 813 – 822 . 10.1007/s00213-006-0686-7 17279377

[b36] Kosten , L. , Verhaeghe , J. , Wyffels , L. , Stroobants , S. , & Staelens , S. ( 2018 ). Acute ketamine infusion in rat does not affect in vivo [11C]ABP688 binding to metabotropic glutamate receptor subtype 5 . Molecular Imaging , 17 , 1536012118788636 . 10.1177/1536012118788636 30213221 PMC6144515

[b37] Larsen , M. , Hegstad , E. , Berg-Johnsen , J. , & Langmoen , I. A. ( 1997 ). Isoflurane increases the uptake of glutamate in synaptosomes from rat cerebral cortex . British Journal of Anaesthesia , 78 ( 1 ), 55 – 59 . 10.1093/bja/78.1.55 9059205

[b38] Larsen , M. , Valo , E. , Berg-Johnsen , J. , & Langmoen , I. ( 1998 ). Isoflurane reduces synaptic glutamate release without changing cytosolic free calcium in isolated nerve terminals . European Journal of Anaesthesiology , 15 ( 2 ), 224 – 229 . 10.1111/j.0265-0215.1998.00275.x 9587730

[b39] Laruelle , M. ( 2012 ). Measuring dopamine synaptic transmission with molecular imaging and pharmacological challenges: The state of the art . In G. Gründer (Ed.), Molecular imaging in the clinical neurosciences. Neuromethods (vol 71 , pp. 163 – 203 ). Humana Press . 10.1007/7657_2012_45

[b40] Laruelle , M. , Iyer , R. N. , al-Tikriti , M. S. , Zea-Ponce , Y. , Malison , R. , Zoghbi , S. S. , Baldwin , R. M. , Kung , H. F. , Charney , D. S. , Hoffer , P. B. , Innis , R. B. , & Bradberry , C. W. ( 1997 ). Microdialysis and SPECT measurements of amphetamine-induced dopamine release in nonhuman primates . Synapse , 25 ( 1 ), 1 – 14 . 10.1002/(sici)1098-2396(199701)25:1<1::aid-syn1>3.0.co;2-h 8987142

[b41] Leuzy , A. , Zimmer , E. R. , Dubois , J. , Pruessner , J. , Cooperman , C. , Soucy , J. P. , Kostikov , A. , Schirmaccher , E. , Désautels , R. , Gauthier , S. , & Rosa-Neto , P. ( 2016 ). In vivo characterization of metabotropic glutamate receptor type 5 abnormalities in behavioral variant FTD . Brain Structure & Function , 221 ( 3 ), 1387 – 1402 . 10.1007/s00429-014-0978-3 25596865

[b42] Levenga , J. , Hayashi , S. , de Vrij , F. M. , Koekkoek , S. K. , van der Linde , H. C. , Nieuwenhuizen , I. , Song , C. , Buijsen , R. A. , Pop , A. S. , Gomezmancilla , B. , Nelson , D. L. , Willemsen , R. , Gasparini , F. , & Oostra , B. A. ( 2011 ). AFQ056, a new mGluR5 antagonist for treatment of fragile X syndrome . Neurobiology of Disease , 42 ( 3 ), 311 – 317 . 10.1016/j.nbd.2011.01.022 21316452

[b43] Lupinsky , D. , Moquin , L. , & Gratton , A. ( 2010 ). Interhemispheric regulation of the medial prefrontal cortical glutamate stress response in rats . Journal of Neuroscience , 30 ( 22 ), 7624 – 7633 . 10.1523/jneurosci.1187-10.2010 20519537 PMC6632388

[b44] Lupinsky , D. , Moquin , L. , & Gratton , A. ( 2017 ). Interhemispheric regulation of the rat medial prefrontal cortical glutamate stress response: Role of local GABA- and dopamine-sensitive mechanisms . Psychopharmacology , 234 ( 3 ), 353 – 363 . 10.1007/s00213-016-4468-6 27822602

[b45] Maeng , S. , Zarate , C. A. , Jr., Du , J. , Schloesser , R. J. , McCammon , J. , Chen , G. , & Manji , H. K. ( 2008 ). Cellular mechanisms underlying the antidepressant effects of ketamine: Role of alpha-amino-3-hydroxy-5-methylisoxazole-4-propionic acid receptors . Biological Psychiatry , 63 ( 4 ), 349 – 352 . 10.1016/j.biopsych.2007.05.028 17643398

[b46] Marquez de Prado , B. , Castaneda , T. R. , Galindo , A. , del Arco , A. , Segovia , G. , Reiter , R. J. , & Mora , F. ( 2000 ). Melatonin disrupts circadian rhythms of glutamate and GABA in the neostriatum of the awake rat: A microdialysis study . Journal of Pineal Research , 29 ( 4 ), 209 – 216 . 10.1034/j.1600-0633.2002.290403.x 11068943

[b47] Martinez , D. , Slifstein , M. , Nabulsi , N. , Grassetti , A. , Urban , N. B. , Perez , A. , Liu , F. , Lin , S.-F. , Ropchan , J. , Mao , X. , Kegeles , L. S. , Shungu , D. C. , Carson , R. E. , & Huang , Y. ( 2014 ). Imaging glutamate homeostasis in cocaine addiction with the metabotropic glutamate receptor 5 positron emission tomography radiotracer [11C]ABP688 and magnetic resonance spectroscopy . Biological Psychiatry , 75 ( 2 ), 165 – 171 . 10.1016/j.biopsych.2013.06.026 24035345 PMC4106018

[b48] Mawlawi , O. , Martinez , D. , Slifstein , M. , Broft , A. , Chatterjee , R. , Hwang , D. R. , Huang , Y. , Simpson , N. , Ngo , K. , Van Heertum , R. , & Laruelle , M. ( 2001 ). Imaging human mesolimbic dopamine transmission with positron emission tomography: I. Accuracy and precision of D(2) receptor parameter measurements in ventral striatum . Journal of Cerebral Blood Flow and Metabolism , 21 ( 9 ), 1034 – 1057 . 10.1097/00004647-200109000-00002 11524609

[b49] Michele , S. M. , Minuzzi , L. , Benkelfat , C. , Soucy , J.-P. , Kirlow , A. , Schirrmacher , E. , Angle , M. , Verhaeghe , J. A. J. , Massarweh , G. , Reader , A. J. , Aliaga , A. , Peixoto-Santos , J. E. , Guiot , M.-C. , Kobayashi , E. , Rosa-Neto , P ., & Leyton , M. ( 2024 ). Quantification of [ ^11^ C]ABP688 binding in human brain using cerebellum as reference region: Biological interpretation and limitations . medRxiv , 2024.2002.2012.24302279. 10.1101/2024.02.12.24302279 PMC12272086 39976038

[b50] Milella , M. S. , Marengo , L. , Larcher , K. , Fotros , A. , Dagher , A. , Rosa-Neto , P. , Benkelfat , C. , & Leyton , M. ( 2014 ). Limbic system mGluR5 availability in cocaine dependent subjects: A high-resolution PET [11C]ABP688 study . Neuroimage , 98 , 195 – 202 . 10.1016/j.neuroimage.2014.04.061 24795154

[b51] Miyazaki , H. , Nakamura , Y. , Arai , T. , & Kataoka , K. ( 1997 ). Increase of glutamate uptake in astrocytes: A possible mechanism of action of volatile anesthetics . Anesthesiology , 86 ( 6 ), 1359 – 1366 ; discussion 8A. https://www.ncbi.nlm.nih.gov/pubmed/9197306 9197306 10.1097/00000542-199706000-00018

[b52] Moghaddam , B. , Adams , B. , Verma , A. , & Daly , D. ( 1997 ). Activation of glutamatergic neurotransmission by ketamine: A novel step in the pathway from NMDA receptor blockade to dopaminergic and cognitive disruptions associated with the prefrontal cortex . Journal of Neuroscience , 17 ( 8 ), 2921 – 2927 . 10.1523/jneurosci.17-08-02921.1997 9092613 PMC6573099

[b53] Papp , E. A. , Leergaard , T. B. , Calabrese , E. , Johnson , G. A. , & Bjaalie , J. G. ( 2014 ). Waxholm space atlas of the Sprague Dawley rat brain . Neuroimage , 97 , 374 – 386 . 10.1016/j.neuroimage.2014.04.001 24726336 PMC4160085

[b54] Paxinos , G. , & Watson , C. ( 2005 ). The rat brain in stereotaxic coordinates, 5th edn . Academic Press . 10.1016/b978-0-12-547620-1.50004-7 6110810

[b55] Pitman , R. K. , & Orr , S. P. ( 1986 ). Test of the conditioning model of neurosis: Differential aversive conditioning of angry and neutral facial expressions in anxiety disorder patients . Journal of Abnormal Psychology , 95 ( 3 ), 208 – 213 . 10.1037//0021-843x.95.3.208 3745641

[b56] Pruessner , J. C. , Kirschbaum , C. , Meinlschmid , G. , & Hellhammer , D. H. ( 2003 ). Two formulas for computation of the area under the curve represent measures of total hormone concentration versus time-dependent change . Psychoneuroendocrinology , 28 ( 7 ), 916 – 931 . 10.1016/s0306-4530(02)00108-7 12892658

[b57] Ramadan , S. , Lin , A. , & Stanwell , P. ( 2013 ). Glutamate and glutamine: A review of in vivo MRS in the human brain . NMR in Biomedicine , 26 ( 12 ), 1630 – 1646 . 10.1002/nbm.3045 24123328 PMC3849600

[b58] Saal , D. , Dong , Y. , Bonci , A. , & Malenka , R. C. ( 2003 ). Drugs of abuse and stress trigger a common synaptic adaptation in dopamine neurons . Neuron , 37 ( 4 ), 577 – 582 . 10.1016/S0896-6273(03)00021-7 12597856

[b59] Scala , S. G. , Smart , K. , Cox , S. M. L. , Benkelfat , C. , & Leyton , M. ( 2021 ). PET imaging of type 5 metabotropic glutamate receptors . In Olive , M. F. , Burrows , B. T. , & Leyrer-Jackson , J. M. (Eds.), Metabotropic glutamate receptor technologies (pp. 39 – 56 ). Springer US . 10.1007/978-1-0716-1107-4_3

[b60] Shafiei , G. , Zeighami , Y. , Clark , C. A. , Coull , J. T. , Nagano-Saito , A. , Leyton , M. , Dagher , A. , & Misic , B. ( 2019 ). Dopamine signaling modulates the stability and integration of intrinsic brain networks . Cerebral Cortex , 29 ( 1 ), 397 – 409 . 10.1093/cercor/bhy264 30357316 PMC6294404

[b61] Sheth , C. , Prescot , A. P. , Legarreta , M. , Renshaw , P. F. , McGlade , E. , & Yurgelun-Todd , D. ( 2019 ). Reduced gamma-amino butyric acid (GABA) and glutamine in the anterior cingulate cortex (ACC) of veterans exposed to trauma . Journal of Affective Disorders , 248 , 166 – 174 . 10.1016/j.jad.2019.01.037 30735853

[b62] Shigemoto , R. , Nomura , S. , Ohishi , H. , Sugihara , H. , Nakanishi , S. , & Mizuno , N. ( 1993 ). Immunohistochemical localization of a metabotropic glutamate receptor, mGluR5, in the rat brain . Neuroscience Letters , 163 ( 1 ), 53 – 57 . 10.1016/0304-3940(93)90227-c 8295733

[b63] Smart , K. , Scala , S. G. , El Mestikawy , S. , Benkelfat , C. , & Leyton , M. ( 2017 ). Cocaine addiction and mGluR5 . In Preedy , V. R. (Ed.), The neuroscience of cocaine (pp. 269 – 278 ). Academic Press . 10.1016/b978-0-12-803750-8.00027-0

[b64] Spielberger , C. D. G. , & Gorsuch , R. L. ( 1983 ). Manual for the state-trait anxiety inventory (form Y) (“self-evaluation questionnaire”) . Consulting Psychologists Press . http://hdl.handle.net/10477/1873

[b65] Stuber , G. D. , Hopf , F. W. , Hahn , J. , Cho , S. L. , Guillory , A. , & Bonci , A. ( 2008 ). Voluntary ethanol intake enhances excitatory synaptic strength in the ventral tegmental area . Alcoholism, Clinical and Experimental Research , 32 ( 10 ), 1714 – 1720 . 10.1111/j.1530-0277.2008.00749.x 18627359 PMC3040033

[b66] Ullmann , E. , Chrousos , G. , Perry , S. W. , Wong , M.-L. , Licinio , J. , Bornstein , S. R. , Tseilikman , O. , Komelkova , M. , Lapshin , M. S. , Vasilyeva , M. , Zavjalov , E. , Shevelev , O. , Khotskin , N. , Koncevaya , G. , Khotskina , A. S. , Moshkin , M. , Cherkasova , O. , Sarapultsev , A. , Ibragimov , R. , … Yehuda , R. ( 2020 ). Offensive behavior, striatal glutamate metabolites, and limbic–hypothalamic–pituitary–adrenal responses to stress in chronic anxiety . International Journal of Molecular Sciences , 21 ( 20 ). 10.3390/ijms21207440 PMC7589759 33050201

[b67] Vigneault , E. , Poirel , O. , Riad , M. , Prud'homme , J. , Dumas , S. , Turecki , G. , Fasano , C. , Mechawar , N. , & El Mestikawy , S. ( 2015 ). Distribution of vesicular glutamate transporters in the human brain . Frontiers in Neuroanatomy , 9 , 23 . 10.3389/fnana.2015.00023 25798091 PMC4350397

[b68] Wyss , M. T. , Ametamey , S. M. , Treyer , V. , Bettio , A. , Blagoev , M. , Kessler , L. J. , Burger , C. , Weber , B. , Schmidt , M. , Gasparini , F. , & Buck , A. ( 2007 ). Quantitative evaluation of 11C-ABP688 as PET ligand for the measurement of the metabotropic glutamate receptor subtype 5 using autoradiographic studies and a beta-scintillator . Neuroimage , 35 ( 3 ), 1086 – 1092 . 10.1016/j.neuroimage.2007.01.005 17320417

[b69] Yuksel , C. , & Ongur , D. ( 2010 ). Magnetic resonance spectroscopy studies of glutamate-related abnormalities in mood disorders . Biological Psychiatry , 68 ( 9 ), 785 – 794 . 10.1016/j.biopsych.2010.06.016 20728076 PMC2955841

[b70] Yushkevich , P. A. , Piven , J. , Hazlett , H. C. , Smith , R. G. , Ho , S. , Gee , J. C. , & Gerig , G. ( 2006 ). User-guided 3D active contour segmentation of anatomical structures: Significantly improved efficiency and reliability . Neuroimage , 31 ( 3 ), 1116 – 1128 . 10.1016/j.neuroimage.2006.01.015 16545965

[b71] Zimmer , E. R. , Parent , M. J. , Leuzy , A. , Aliaga , A. , Aliaga , A. , Moquin , L. , Schirrmacher , E. S. , Soucy , J.-P. , Skelin , I. , Gratton , A. , Gauthier , S. , & Rosa-Neto , P. ( 2015 ). Imaging in vivo glutamate fluctuations with [11C]ABP688: A GLT-1 challenge with ceftriaxone . Journal of Cerebral Blood Flow and Metabolism , 35 ( 7 ), 1169 – 1174 . 10.1038/jcbfm.2015.35 25806702 PMC4640271

